# Targeted Changes of the Cell Wall Proteome Influence *Candida albicans* Ability to Form Single- and Multi-strain Biofilms

**DOI:** 10.1371/journal.ppat.1004542

**Published:** 2014-12-11

**Authors:** Vitor Cabral, Sadri Znaidi, Louise A. Walker, Hélène Martin-Yken, Etienne Dague, Mélanie Legrand, Keunsook Lee, Murielle Chauvel, Arnaud Firon, Tristan Rossignol, Mathias L. Richard, Carol A. Munro, Sophie Bachellier-Bassi, Christophe d'Enfert

**Affiliations:** 1 Institut Pasteur, Unité Biologie et Pathogénicité Fongiques, Département Génomes et Génétique, Paris, France; 2 INRA, USC2019, Paris, France; 3 Univ. Paris Diderot, Sorbonne Paris Cité, Cellule Pasteur, Paris, France; 4 School of Medical Sciences, University of Aberdeen, Aberdeen, United Kingdom; 5 INSA, UPS, INP, ISAE, LAAS, Université de Toulouse, Toulouse, France; 6 UMR792 Ingénierie des Systèmes Biologiques et des Procédés, INRA, Toulouse, France; 7 UMR5504, CNRS, Toulouse, France; 8 LAAS, CNRS, Toulouse, France; 9 INRA, UMR1319 Micalis, Jouy-en-Josas, France; 10 AgroParisTech, UMR Micalis, Thiverval Grignon, France; Carnegie Mellon University, United States of America

## Abstract

Biofilm formation is an important virulence trait of the pathogenic yeast *Candida albicans*. We have combined gene overexpression, strain barcoding and microarray profiling to screen a library of 531 *C. albicans* conditional overexpression strains (∼10% of the genome) for genes affecting biofilm development in mixed-population experiments. The overexpression of 16 genes increased strain occupancy within a multi-strain biofilm, whereas overexpression of 4 genes decreased it. The set of 16 genes was significantly enriched for those encoding predicted glycosylphosphatidylinositol (GPI)-modified proteins, namely Ihd1/Pga36, Phr2, Pga15, Pga19, Pga22, Pga32, Pga37, Pga42 and Pga59; eight of which have been classified as pathogen-specific. Validation experiments using either individually- or competitively-grown overexpression strains revealed that the contribution of these genes to biofilm formation was variable and stage-specific. Deeper functional analysis of *PGA59* and *PGA22* at a single-cell resolution using atomic force microscopy showed that overexpression of either gene increased *C. albicans* ability to adhere to an abiotic substrate. However, unlike *PGA59*, *PGA22* overexpression led to cell cluster formation that resulted in increased sensitivity to shear forces and decreased ability to form a single-strain biofilm. Within the multi-strain environment provided by the *PGA22*-non overexpressing cells, *PGA22*-overexpressing cells were protected from shear forces and fitter for biofilm development. Ultrastructural analysis, genome-wide transcript profiling and phenotypic analyses in a heterologous context suggested that *PGA22* affects cell adherence through alteration of cell wall structure and/or function. Taken together, our findings reveal that several novel predicted GPI-modified proteins contribute to the cooperative behaviour between biofilm cells and are important participants during *C. albicans* biofilm formation. Moreover, they illustrate the power of using signature tagging in conjunction with gene overexpression for the identification of novel genes involved in processes pertaining to *C. albicans* virulence.

## Author Summary

*Candida albicans* is the most prevalent human fungal pathogen. Its ability to cause disease relies, in part, on the formation of biofilms, a protective structure of highly adherent cells tolerant to antifungal agents and the host immune response. The biofilm is considered as a persistent root of infection, disseminating infectious cells to other locations. In this study, we performed large-scale phenotypic analyses aimed at identifying genes whose overexpression affects biofilm development in *C. albicans*. Our screen relied on a collection of 531 *C. albicans* strains, each conditionally overexpressing one given gene and carrying one specific molecular tag allowing the quantification of strain abundance in mixed-population experiments. Our results strikingly revealed the enrichment of strains overproducing poorly-characterized surface proteins called Pgas (*P*utative *G*PI-*A*nchored proteins), within a 531-strain-containing biofilm model. We show that these *PGA* genes differentially contribute to single-strain and multi-strain biofilm formation and are involved in specific stages of the biofilm developmental process. Taken together, our results reveal the importance of *C. albicans* cell surface proteins during biofilm formation and reflect the powerful use of strain barcoding in combination with gene overexpression to identify genes and/or pathways involved in processes pertaining to virulence of pathogenic microbes.

## Introduction

*Candida albicans* is the most predominant human fungal pathogen, causing both superficial and hematogenously disseminated infections [1]. These infections are complicated by *C. albicans'* ability to form biofilms, which are complex three-dimensional microbial structures attached to either biotic or abiotic surfaces and encased in an extracellular matrix [2]–[5]. Biofilms play a crucial role in *C. albicans* virulence as they result in decreased susceptibility to both antimicrobial agents and the host immune system [2], [5]–[7]. *C. albicans* biofilms are composed of yeast and hyphal cells, and the ability to switch between these morphotypes is essential for normal biofilm formation [8]–[10]. Additional understanding of the mechanisms of biofilm formation in *C. albicans* has been gained over recent years with the discovery of various regulators and effectors involved in this process (reviewed in [11]). In this respect, several cell wall proteins have been shown to play crucial roles during biofilm formation. For instance, the Bcr1 transcription factor, required for biofilm formation, was shown to control the expression of genes encoding cell wall proteins, among which the *ALS3*, *ALS1*, and *HWP1* genes contribute to biofilm formation and integrity [12]–[14]. Heterotypic interactions between Als1 and Als3, members of the Als family of glycophosphatidylinositol (GPI)-anchored agglutinin-like cell wall proteins, and the hyphal wall protein Hwp1, appear crucial for cell-cell interactions within biofilms [15]. Other GPI-anchored proteins play positive or negative roles at different stages of biofilm formation, such as Ywp1 (Pga24), Eap1 (Pga47), Pga26, Pga1, and members of the CFEM family (Pga10, Rbt5 and Csa1) [16]–[21].

To date, the investigation of molecular determinants of biofilm formation in *C. albicans* has largely relied on phenotypic analyses of loss-of-function mutants for genes predicted to play a role in this process, based on their expression profile, function or cellular location [12], [22]–[25]. Gene overexpression is an alternative strategy for studying gene function. It mimics gain-of-function mutations, provides a complement to loss-of-function phenotypes and allows the role of both essential and non-essential genes or individual genes within multi-gene families to be studied [26]. Gene overexpression has been successfully used in *Saccharomyces cerevisiae* to reveal new signalling pathways [27] and identify transcription factor targets [28]. More recently, overexpression approaches in *C. albicans* identified genes involved in fitness, adherence, morphogenesis, pheromone response and antifungal resistance [29]–[33] as well as the characterization of transcription factor targets [13], [23], [34]. To date, the largest collection of overexpression plasmids that exists for *C. albicans* genes has been developed in our laboratory [30]. This collection includes 337 uniquely barcoded plasmids allowing tetracycline-inducible overexpression of genes encoding components of signalling networks, in particular protein kinases, protein phosphatases and transcription factors [30].

Here, we have extended this plasmid collection to include genes encoding *C. albicans* predicted cell wall proteins and genes involved in genome dynamics. We took advantage of the molecular barcoding of the cognate overexpression strains to develop a signature-tagged overexpression (ST-OE) screen aimed at identifying genes whose overexpression affects fitness during planktonic cell growth and/or biofilm development in *C. albicans*. Our results specifically highlight the impact of overexpressing specific cell surface GPI-modified proteins on the ability of *C. albicans* to form single- or multi-strain biofilms and reveal their role in adhesion and/or cell-cell interactions.

## Results

### Construction of a signature-tagged *C. albicans* overexpression collection

**Figure 1:**
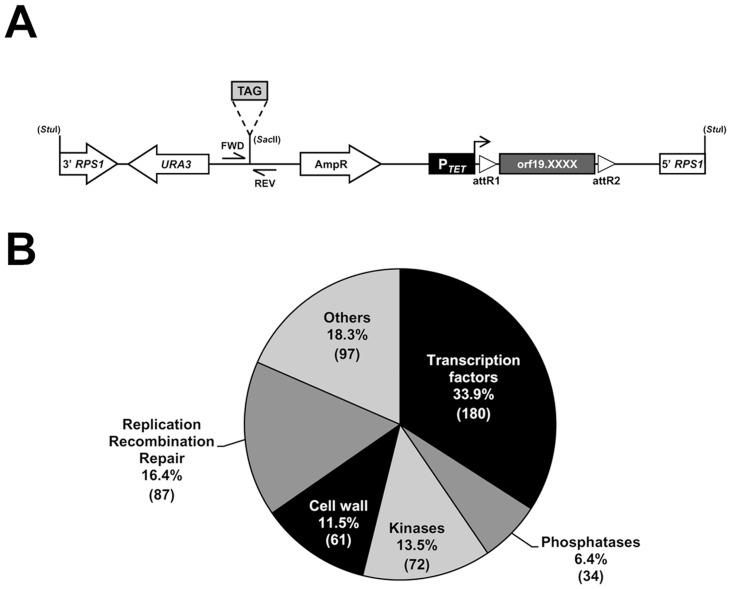
Construction of a barcoded tetracycline-inducible overexpression strain collection. (A) Schematic representation of the *Stu*I-linearized, signature-tagged (TAG), overexpression vector CIp10-P*_TET_*-GTW that was used to create the 531-strain collection. Expression of each of the 531 ORFs (orf19.XXXX; dark gray box) is under the control of the tetracycline-inducible promoter (P*_TET_*, black box), which is activated in the presence of doxycycline (horizontal arrow). The barcoded overexpression cassette is integrated at the *RPS1* locus following a *Stu*I digestion. The ORF is flanked by the lambda phage attachment sequences R1 and R2 (open triangles, attR1 and attR2) that allow recombination-mediated transfer of ORFs from an entry vector collection to the destination barcoded vectors. Transformant selection markers are depicted with open arrows. Tag amplification using forward (FWD) and reverse (REV) primers (CIP-SAC2-UP-2and CIP-SAC2-DWN-2, S6 Table) allows quantification of strain abundance in mixed population experiments. (B) Pie chart of the functional categories of the 531 ORFs included in the collection. The number of ORFs is indicated between parentheses.

We generated a collection of signature-tagged *C. albicans* conditional overexpression strains using partial *C. albicans* ORFeome libraries ([30]; Legrand *et al.*, in preparation; Walker *et al*., in preparation) and a collection of barcoded derivatives of the conditional overexpression plasmid CIp10-P*_TET_*-GTW ([30]; Fig. 1A, See Materials and Methods for details). The resulting collection includes 531 strains conditionally overexpressing individual genes encoding (or predicted to encode) transcription factors (180 ORFs), protein kinases (72 ORFs), protein phosphatases (34 ORFs), proteins related to DNA replication, recombination and repair (87 ORFs), predicted cell surface proteins (61 ORFs) and others (Fig. 1B; S1 Table for details).

We tested the efficiency of detecting each of the 531 tagged strains from a pool with equal strain representation using an oligonucleotide microarray carrying both forward and reverse probe sequences for every barcode (S1 Figure and S1 Table, see Materials and Methods). The microarrays efficiently detected nearly 90% of the pooled strains with only 11.6% of total strain-matching probes (123 out of 1062) having log_2_-transformed Cy5 and Cy3 signal intensities lower than 8 (S1 Figure), suggesting that just over 10% were underrepresented or that the corresponding tags had low hybridization efficiency. Strain detection with the microarrays was highly reproducible, as shown by the high Pearson correlation coefficients between 2 independent pool replicates (S1B Figure).

### Identification of *C. albicans* genes whose overexpression alters cell fitness during planktonic growth

**Figure 2:**
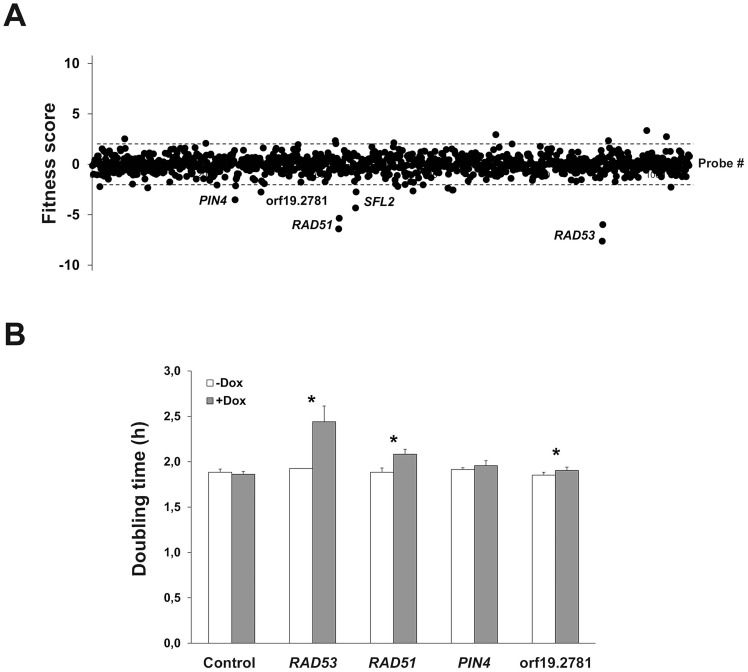
Identification of genes whose overexpression alters planktonic cell fitness. (A) The effect of gene overexpression on cell growth was tested in GHAUM medium at 30°C in the absence or presence of 50 µg.mL^−1^ doxycycline for 16 generations. The experiment was conducted twice independently (n = 2 biological replicates). Samples were subjected to genomic DNA extraction followed by PCR-amplification of the barcodes, indirect fluorescent dye labeling (Dox-treated sample: Cy5-labeled; untreated control: Cy3-labeled) and hybridization to a barcode microarray with both forward and reverse complemented probes for every barcode (two black circles next to each gene name/orf19 nomenclature represent forward and reverse complemented probe sequences). Fitness scores (Z-score for each tag) are shown on the *y*-axis. The corresponding probe number ranked using the orf19 nomenclature in ascending order is shown on the *x*-axis. Z-score calculations were performed using Arraypipe v2.0. Dashed lines correspond to the Z-score values +2.0 (upper line) and −2.0 (lower line). Names or orf19 nomenclature of some of the genes whose overexpression alters strain fitness are shown. (B) Confirmation of the microarray data by liquid growth assay of strains overexpressing *RAD53*, *RAD51*, *PIN4*, and *ORF19.2781* grown three times independently. Doubling time in hours (mean from n = 3 and error bars denote standard deviations) is indicated on the *y*-axis for each strain in the absence (white bar) or presence (gray bar) of 50 µg.mL^−1^ doxycycline (Dox); statistical significance was assigned (p≤0.05, asterisks) by performing 2-tailed Student's *t*-tests and assuming groups of equal variances.

As a proof-of-principle for the ST-OE approach, we evaluated the impact of overexpressing each of the 531 genes on planktonic strain fitness. Pooled strains were grown for 16 generations in GHAUM medium (see Materials and Methods) at 30°C in the presence or absence of 50 µg.mL^-1^ doxycycline (Dox), with dilution in fresh medium after 8 generations to avoid saturation of the culture. Genomic DNA was prepared, followed by barcode amplification, labeling with Cy3 (untreated) or Cy5 (Dox-treated) dyes and hybridization to the barcode microarray. We found that overexpression of 5 genes, namely *RAD53*, *RAD51*, *PIN4, SFL2* and *ORF19.2781*, decreased strain fitness using a Z-score (i.e. number of standard deviations from the population mean) cutoff of −2.0 (Fig. 2A and S2 Table). Notably, we failed to detect any gene conferring increased fitness (Fig. 2A). Liquid growth assays of individual strains grown independently confirmed an increased doubling time in Dox-treated cells overexpressing *RAD53*, *RAD51* and *ORF19.2781*, relative to untreated cells (Fig. 2B), in agreement with our microarray data. As morphology alterations affect turbidity measurements, the *SFL2*-overexpressing strain which shows extensive filamentation in overexpression conditions [30], [35]–[37] was omitted from this assay. Taken together, these data indicated that our ST-OE approach could be used to identify genes whose overexpression affects *C. albicans* fitness.

### Identification of *C. albicans* genes whose overexpression alters strain abundance within a multi-strain biofilm

**Figure 3:**
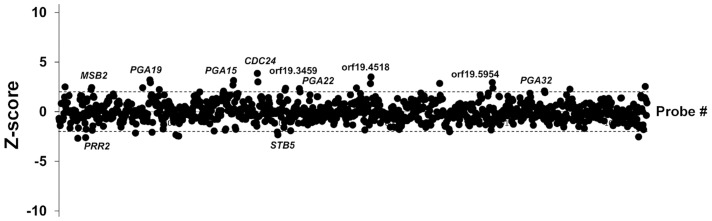
Competitive strain occupancy profiling in a multi-strain biofilm. Z-scores for every barcode (both forward and reverse complemented probes) are indicated on the *y*-axis and the corresponding tag is indicated on the *x*-axis (ranked according to ascending orf19 nomenclature). Positive values indicate increased occupancy within the multi-strain biofilm, whereas negative values indicate decreased occupancy. Dashed lines indicate Z-scores of 2.0 (upper dashed line) and −2.0 (lower dashed line). For simplicity, only genes whose overexpression affects strain occupancy by at least two standard deviations (Z-scores >2.0 or <−2.0) are labeled.

We used the ST-OE approach to identify *C. albicans* genes whose overexpression alters strain abundance within a multi-strain biofilm. To this end, the 531-strain pool was grown overnight in GHAUM medium at 30°C in the presence or absence of 50 µg.mL^−1^ doxycycline. Cells were allowed to adhere to Thermanox slides that were subsequently incubated at 37°C for 40 h in a microfermentor under a continuous flow of GHAUM medium in the presence or absence of 50 µg.mL^−1^ doxycycline. Genomic DNA was extracted from the resulting mature biofilms followed by barcode amplification, Cy3 (untreated)/Cy5 (Dox-treated) labeling and hybridization to barcode microarrays. The entire experiment was performed using 8 biological replicates and two independent analyses of our microarray data were performed (See Materials and Methods for details). We found 20 genes whose overexpression altered strain abundance in the multi-strain biofilm. Only one gene (*ORF19.2781*) was common to both planktonic and biofilm data, suggesting that the remaining 19 genes were not linked to a growth defect (Table 1, Fig. 3 and S3 Table). Overexpression of 16 out of these 20 genes resulted in increased strain abundance within the multi-strain biofilm (Table 1). A gene ontology (GO) term enrichment analysis was performed using the GO Term Finder at the *Candida* Genome Database [38]–[40] and the 531 gene set as a reference. Strikingly, among the 20 identified genes we found a significant enrichment of the GO term “cell wall” (*P* = 1.4×10^−5^) including 10 genes encoding (or predicted to encode) proteins involved in cell wall biogenesis and integrity (*IHD1/PGA36, PGA15, PGA19, PGA22, PGA32, PGA37, PGA42, PGA59, PHR2* and *TOS1*, Table 2). Similarly, when the enrichment analysis was performed using broad functional categories, we observed a significant enrichment for cell surface protein-encoding genes (*P = *1.31×10^−5^; Table 2). Overexpression of these 10 genes did not significantly promote or inhibit morphogenesis when compared to strain SC5314 (S2A Figure). Moreover, their overexpression did not significantly impact growth rates as judged by colony size on solid GHAUM medium at 30°C and 37°C (S2B Figure). Thus, our data suggested that the increased occupancy of the multi-strain biofilm by the selected strains was not a consequence of altering growth rate or morphogenesis.

**Table 1:** List of genes whose overexpression affects strain occupancy in a multi-strain biofilm.

Orf number	Gene Name	Fold change^1^	Z-score^2^	Occupancy^3^
*ORF19.3174*	*CDC24*	4.81	3.87	+
*ORF19.2033*	*PGA19*	4.09	3.2	+
*ORF19.2878*	*PGA15*	3.47	3.15	+
*ORF19.1490*	*MSB2*	3.23	2.44	+
*ORF19.4518*	*-*	3.75	3.51	+
*ORF19.3738*	*PGA22*	2.58	2.33	+
*ORF19.6784*	*PGA32*	2.45	2.09	+
*ORF19.2767*	*PGA59*	3.11	2.08	+
*ORF19.5954*	*-*	3.45	2.95	+
*ORF19.6573*	*BEM2*	2.08	1.86	+
*ORF19.5760*	*IHD1*	2.34	1.71	+
*ORF19.2907*	*PGA42*	2.21	1.84	+
*ORF19.6081*	*PHR2*	2.13	1.56	+
*ORF19.3459*	*-*	3.12	2.37	+
*ORF19.1690*	*TOS1*	2.06	1.59	+
*ORF19.3923*	*PGA37*	2.18	1.52	+
*ORF19.5343*	*ASH1*	−2.21	−2.02	−
*ORF19.2781*	*-*	−2.23	−1.86	−
*ORF19.3308*	*STB5*	−2.70	−2.33	−
*ORF19.1341*	*PRR2*	−2.95	−2.6	−

1 Fold Change in strain occupancy between Dox-treated and -untreated biofilm as deduced from GeneSpring GX 11 analysis; all strains showed p≤0.05. The fold-change with a higher value among 2 replicate probes is shown (S3 Table, sheet named “GeneSpring analysis-hits p<0.05”).

2 Z-score obtained from Arraypipe v2.0 analysis of data for Dox-treated and -untreated biofilm; all strains showed p≤0.05. The Z-score with a higher value among 2 replicate probes is shown (S3 Table, sheet named “Complete Biofilm Data-Arraypipe”).

3 +: increased representation of the overexpression strain in the Dox-treated multi-strain biofilm relative to the untreated multi-strain biofilm; -: decreased representation of the overexpression strain in the Dox-treated multi-strain biofilm relative to the untreated multi-strain biofilm.

**Table 2:** Distribution of genes across categories in the strain collection and the gene set selected through the signature-tagged overexpression screen.

Functional categories	Strain collection		Strains with increased or decreased biofilm occupancy upon gene overexpression		p-value^1^
	number of strains	%	number of strains	%	
Protein Kinases	72	13.5	4	20.0	0.16
Protein Phosphatases	34	6.4	0	0.0	0.26
Replication, Recombination & Repair	87	16.4	0	0.0	0.03
Cell Surface Proteins	61	11.5	10	50.0	1.31×10^−5^
Transcription Factors	180	33.9	2	10.0	0.01
*Others*	97	18.3	4	20.0	0.22
**Total**	**531**		**20**		

1 p-values were calculated using a hypergeometric test

### Impact of overexpressing selected genes on single-strain biofilm formation

**Figure 4:**
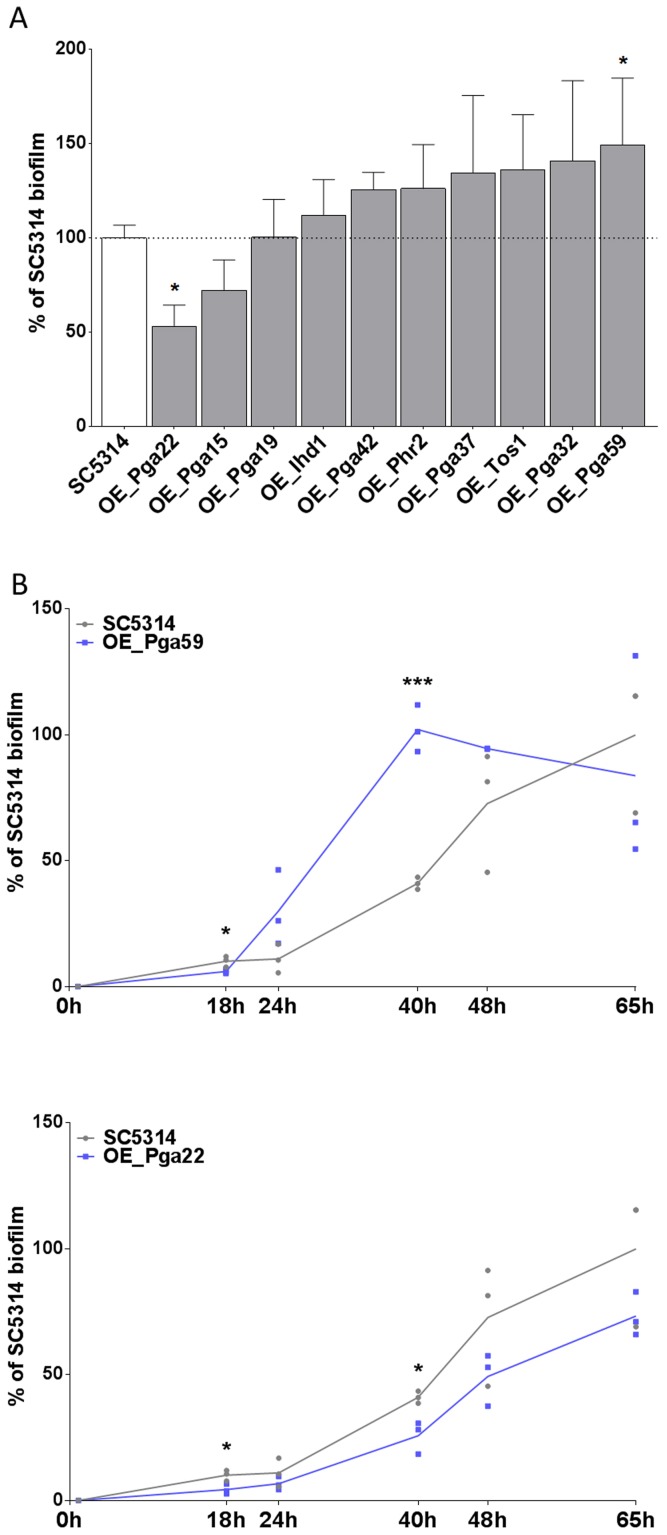
Overexpression of selected cell wall protein genes impacts the kinetics of biofilm formation. (A) Biofilms of overexpression strains and the SC5314 control strain were developed in a microfermentor under a continuous flow of GHAUM medium in the presence of doxycycline (50 µg.mL^−1^) and their dry weight was measured after 40 h. The dry mass of the biofilms are expressed in percent of the average dry mass of biofilms formed by the control strain SC5314. Data are average of at least 3 replicates and standard errors of means are shown. Significance of the weight differences relative to the control strain biofilm weight were assessed using Student's *t-*test. * p≤0.05. (B) Biofilms of overexpression strains and the SC5314 control strain were developed in a microfermentor under a continuous flow of GHAUM medium in the presence of doxycycline (50 µg.mL^−1^) and their dry weight was measured after 0, 18, 24, 40, 48, and 65 h. The dry mass of the biofilms are expressed as percentage of the average dry mass of 65 h biofilms formed by the control strain SC5314. Data points corresponding to biological replicates are shown in black (SC5314 control strain) or blue [overexpression strains: (B) Top panel, *PGA59*; Bottom panel, *PGA22*].

The selected overexpression strains were tested for their ability to form single strain biofilms under conditions similar to those used for our mixed-population screen. Biofilms were grown in the continuous-flow microfermentor system. The biomass obtained after 40 h of biofilm growth was quantified and compared to the biomass formed by the wild type strain SC5314 grown under the same conditions. As shown in Fig. 4A, the strain overexpressing *PGA59* displayed increased biomass, consistent with our mixed-population data. Decreased biofilm biomass was observed upon *PGA22* overexpression (Fig. 4A), contrasting with the positive impact of overexpressing this gene on strain abundance in the multi-strain biofilm (Fig. 3 and Table 1). Finally, overexpression of the remaining genes did not significantly affect biofilm biomass (Fig. 4A).

We further compared biofilm formation of the wild-type and the *PGA59-* and *PGA22-*overexpression strains during a kinetic experiment in the continuous-flow fermentor system under inducing conditions. The biofilm biomass was quantified at 18, 24, 40, 48, and 65 h of biofilm growth. The overexpression strains behaved similarly until the first time-point (18 h; Fig. 4B). The *PGA59-*overexpression strain formed a biofilm faster than the remaining strains from 18 h onwards (Fig. 4B, upper panel). In contrast, the *PGA22-*overexpression strain developed into biofilms more slowly than the wild-type strain (Fig. 4B, lower panel). Overall, these results were consistent with those obtained in the end-point analysis (Fig. 4A) and suggested that the behavior of the *PGA22-*overexpression strain differed when grown in single- or multi-strain biofilms.

### Competitive growth of the *PGA22* or *PGA59* overexpression strains results in higher abundance of the corresponding strains in a mixed population biofilm

**Figure 5:**
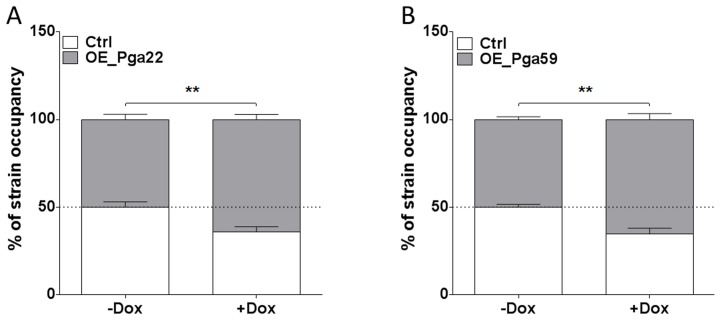
Higher occupancy of strains overexpressing *PGA22* and *PGA59* in a mixed biofilm formed with a wild-type strain. Biofilms were developed for 40 h using as an inoculum mixture with a ratio of 1∶1 BFP-expressing control strain and either a GFP-expressing *PGA22-*overexpressing strain (A) or a GFP-expressing *PGA59-*overexpressing strain (B) in the absence (-Dox) or presence (+ Dox) of 50 µg.mL^−1^ doxycycline. The abundance of each strain in the mixed biofilm was quantified using qPCR of the *GFP* and *BFP* genes. Data was averaged for the 6 replicates and standard error of means are shown; Student's *t-*tests were performed and results are represented on top of each graph (asterisk); ** p≤0.01.

Because of the discrepancy observed for the *PGA22-*overexpression strain in single- or multi-strain biofilms, we assessed whether the increased abundance of *PGA22-* or *PGA59*-overexpression strains in a multi-strain biofilm could be recapitulated in a biofilm formed by either of these strains together with a control strain. The *PGA22-* or *PGA59*-overexpression plasmids were introduced into a *C. albicans* strain that constitutively expressed *GFP* (CEC3781, S4 Table). A strain constitutively expressing *BFP* (CEC3783, S4 Table) was used as a control. A 1∶1 mixture of the *GFP*-tagged *PGA22*- or *PGA59*-overexpressing strains and the *BFP-*expressing control strain was used as an inoculum, and biofilms grown for 40 h in the absence or presence of doxycycline. Genomic DNA was isolated from each biofilm, and strain abundance quantified by qPCR using *GFP* and *BFP* specific primers. In biofilms grown under non-inducing conditions, the overexpression and control strains were equally represented (Fig. 5). By contrast, both *PGA22*- and *PGA59*-overexpression strains were significantly more abundant in the respective biofilms grown under inducing conditions (64% vs. 50% for the *PGA22*-overexpression strain and 65% vs. 50% for the *PGA59*-overexpression strain, with or without doxycycline, respectively; Fig. 5). Consistently, confocal microscopy-acquired fluorescence images showed more GFP-labeled cells relative to BFP cells in the doxycycline-treated mixed biofilms as compared to untreated controls (S3 Figure). A similar increase was observed when *BFP*-expressing *PGA22*- or *PGA59*-overexpression strains were grown in mixed-biofilms with a *GFP*-expressing control strain (S4 Figure). Hence, overexpression of *PGA22* and *PGA59* resulted in increased strain abundance in a mixed biofilm-population.

### *PGA22* and *PGA59* overexpression increases cell adherence

**Figure 6:**
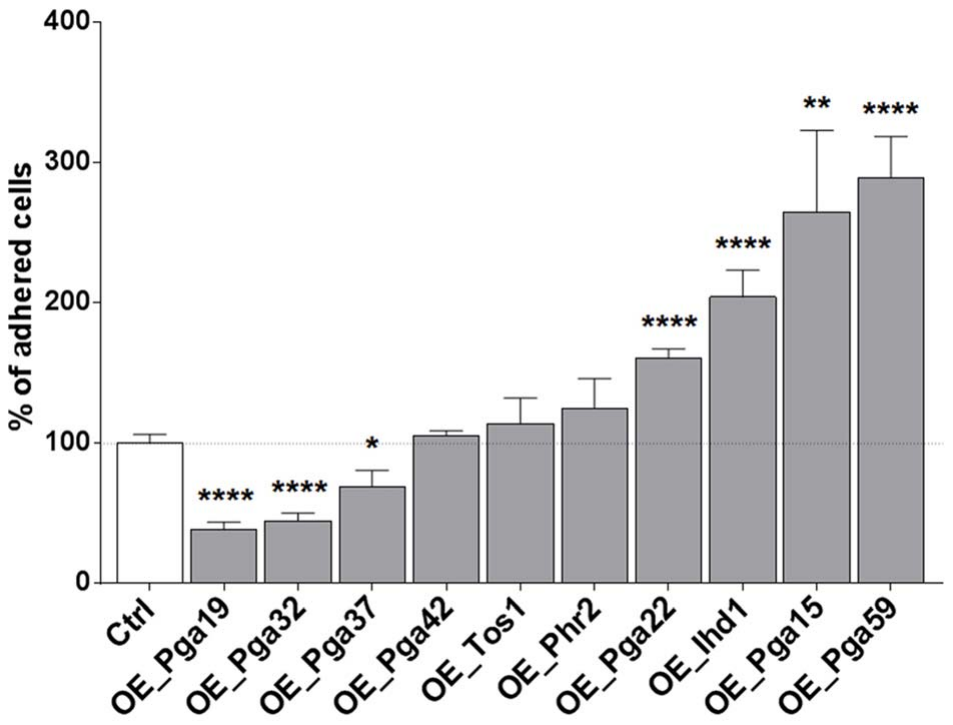
Overexpression of selected cell wall protein genes impacts adherence to Thermanox. Adherence of the overexpression strains to Thermanox was quantified following growth in the absence or presence of 50 µg.mL^−1^ doxycycline. Data were normalized using as reference the adherence shown by each strain in the absence of overexpression. Significance of the adherence differences relative to the control strain adherence were assessed using Student's *t-*tests performed on at least 10 pictures for each strain and results are represented on top of each bar (* p≤0.05, ** p≤0.01, and **** p≤0.0001)

Biofilm formation is a multi-stage process that is initiated upon adherence of *C. albicans* yeast cells to a substrate and reinforced through cell-to-cell interactions (reviewed in [3]). Differences observed for the *PGA22-* and *PGA59-*overexpression strains upon single- or multi-strain biofilm formation might reflect differences in adherence to the substrate or to other biofilm cells. Therefore, strains overexpressing *PGA22* or *PGA59* as well as the other cell wall-related genes identified in our screen were individually tested for their ability to adhere to Thermanox following overnight growth in GHAUM medium at 30°C in the presence or absence of doxycycline. Modest, non-significant, variations in the adherence of the ten strains were observed when they were grown under non-inducing conditions (S5 Figure). In contrast, overexpression of *IHD1/PGA36, PGA15, PGA22* and *PGA59* significantly increased adherence of *C. albicans* to Thermanox, while overexpression of *PGA19*, *PGA32* and *PGA37* decreased it (Fig. 6). The increased adherence phenotype was substrate-independent, as shown in an adhesion assay of the *PGA22*- and *PGA59*-overexpressing strains using a polystyrene substrate (microtiter plate, S6 Figure).

### *PGA22* overexpression results in increased sensitivity of adhered cells to shear forces

**Figure 7:**
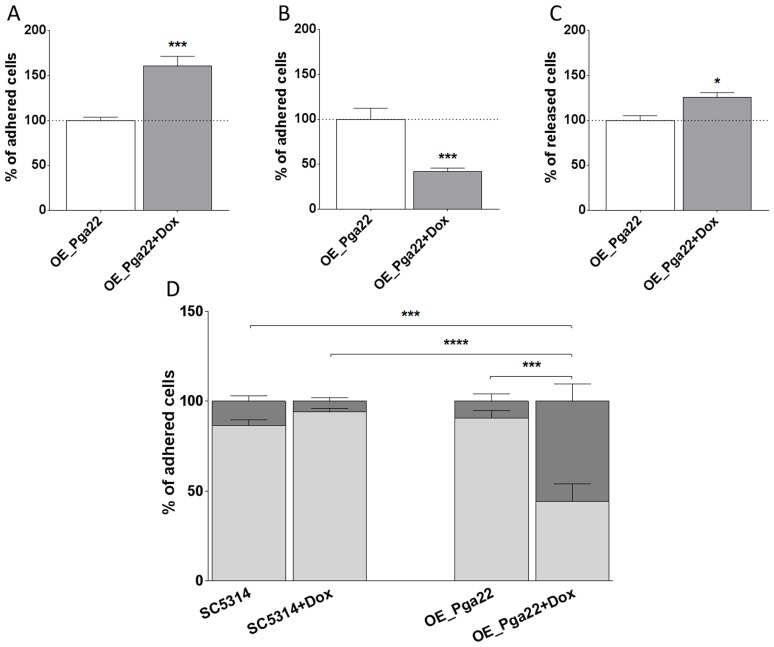
*PGA22-*overexpression triggers cell clustering and increases the sensitivity to shear forces of cells adhered to Thermanox. (A) Percentage of cells adhered to Thermanox at t = 0 h in overexpression-inducing conditions (50 µg.mL^−1^ doxycycline; + Dox, gray bar) relative to non-inducing conditions (-Dox, white bar). Average data of 6 replicates and standard error of means are shown. (B) Percentage of cells adhered to Thermanox following 2 h of biofilm development in overexpression-inducing conditions relative to 2 h of biofilm development in non-inducing conditions; average data of 39 replicates and standard error of means are shown. (C) Percentage of cells released from Thermanox following 2 h of biofilm development in overexpression-inducing conditions relative to 2 h of biofilm development in non-inducing conditions; average data of 3 replicates and standard error of means are shown. (D) Percentage of cells adhered as individual or in small clusters (≤3 cells; light gray bars) or in large clusters (>3 cells; dark gray bars) for the wild-type strain SC5314 and *PGA22*-overexpression strain, in the presence (+ Dox) or absence (-Dox) of doxycycline; average data of 10 replicates and standard error of means are shown. Student's *t-*tests were performed and significant results are represented on the top of each graph (asterisk); * p≤0.05, *** p≤0.001, and **** p≤0.0001.

Results presented above indicated that over-expression of *PGA59* and *PGA22* increased adherence to abiotic surfaces and yet, this could have different outcomes during biofilm formation. While the *PGA59-*overexpression strain showed an increased ability to form biofilms in single- and multi-strain biofilms consistent with increased adherence to the substrate, the *PGA22-*overexpression strain showed decreased or increased ability to form biofilms when grown alone or in combination, respectively. We reasoned that the differences observed for the *PGA22-*overexpression strain might occur at early time points during biofilm development and therefore, examined the fate of cells overexpressing *PGA22* once adhered to Thermanox and exposed to a flow of medium in the microfermentor system. To this aim, the *PGA22-*overexpression strain was grown overnight in the presence or absence of doxycycline, allowed to adhere to Thermanox and incubated in the microfermentor system in the presence or absence of doxycycline for 2 h. Cells attached to the Thermanox at t = 0 h and t = 2 h and those that were released during the 2 h of biofilm growth in the continuous-flow fermentor system were quantified by microscopy or by flow cytometry (see Materials and Methods). At t = 0 h, overexpression of *PGA22* resulted in increased adherence (Fig. 7A); however, at t = 2 h, less cells remained attached to the slide (Fig. 7B), correlating with an increase of released cells during this 2 h period (Fig. 7C). Moreover, careful observation of Thermanox slides under the microscope revealed that upon overexpression of *PGA22*, cells tended to adhere in clusters of more than 3 cells (Fig. 7D). Under a more static environment (*i*.*e*. in the absence of flow), overexpression of *PGA22* did not decrease biofilm formation and showed a tendency to form more biofilm (S7 Figure). In this environment the *PGA59-*overexpression strain still formed more biofilm (S7 Figure). This is in line with the effect of flow in washing out cells adhered to the substrate and consequently affecting biofilm biomass. We further analyzed our confocal microscopy-acquired fluorescence data from the competitive biofilm growth assay under continuous flow of the GFP-labeled strains overexpressing *PGA22* and *PGA59* versus the parental control strain (BFP-labeled, S3 Figure). We quantified abundance of GFP- versus BFP-labeled cells within the bottom layer of the mature biofilm, where early events such as adhesion occur. In the bottom layer of the biofilm, the *PGA22*-overexpressing cells were less abundant as compared to the upper layer (S8 Figure, compare panels A and B). In contrast, *PGA59*-overexpressing cells were more abundant within both the bottom and upper layers of the mature biofilm (S8 Figure, compare panels C and D). Taken together, our results suggested that the apparent increase in adherence of the *PGA22-*overexpression strain was a consequence of increased cell aggregation that rendered adhered cells more susceptible to shear forces occurring in the microfermentor. This may explain why *PGA22* overexpression is detrimental to single-strain biofilm formation but favorable in a potentially protective multi-strain biofilm. In contrast, increased adherence of individual cells of the *PGA59-*overexpression strain to the surface was directly correlated to increased biofilm formation whether alone or in combination.

### *PGA22* and *PGA59* overexpression imparts significant adhesion forces to single *C. albicans* cells exposed to an inert support

**Figure 8:**
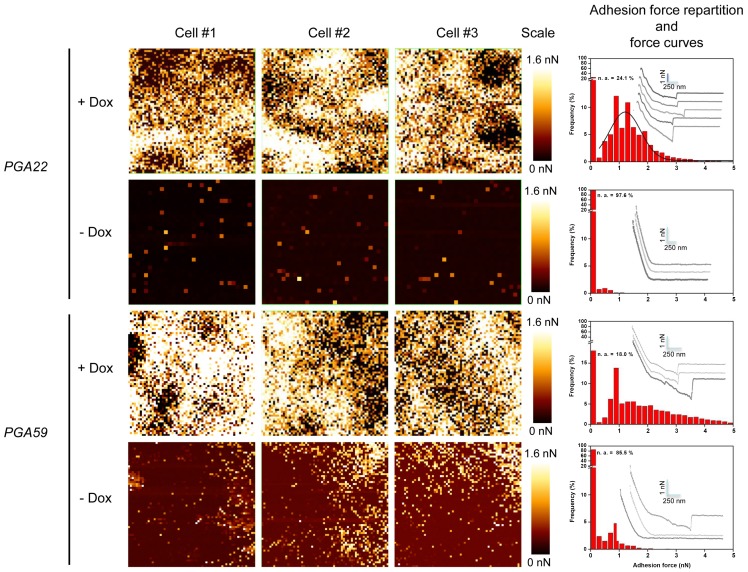
Atomic Force Microscopy-derived adhesion force measurements in single *C. albicans* cells overexpressing *PGA22* and *PGA59*. Adhesion maps in three independent *C. albicans* cells (Cells #1–3, each analyzed area covers 1×1 µm^2^) recorded on *PGA22*- or *PGA59*-overexpression strains treated (+ Dox) or not (-Dox) with 50 µg.mL^−1^ doxycycline during 16 h in YPD. Adhesion scales are shown (Scales; bright yellow, maximum at 1.6 nN; dark red, minimum at 0.0 nN). The corresponding histograms representing the adhesion force repartition (red bars) and force curves (grey lines; scales are indicated with light blue bars) are shown at the right of each panel.

To further investigate the effect of *PGA22* and *PGA59* overexpression on the adherence of single *C. albicans* cells, we performed adhesion force measurements using Atomic Force Microscopy (AFM, Fig. 8). The *PGA22-* and *PGA59*-overexpression strains were grown for 16 h in the presence or absence of 50 µg.mL^−1^ doxycycline in YPD medium and subjected to an adhesion force measurement assay between the AFM tip, composed of Si_3_N_4_, and the cell surface (Fig. 8). We generated adhesion maps (Fig. 8, left panels) where the intensity of each pixel corresponds to the force required to dissociate the AFM tip from the sample (*i*.*e*. adhesion force). Upon doxycycline treatment, adhesion events were detected in 76% of the recorded force curves for *PGA22* overexpression (Fig. 8, + Dox, upper panels), versus less than 3% without treatment (Fig. 8, -Dox, upper panels). We detected only 5% of adhesion events in the recorded force curves on the parental control strain (vector only, S9 Figure). The mean force of *PGA22* overexpression-mediated adhesion events was 1.21 nN±0.55 nN (Fig. 8). The *PGA59*-overexpressing cells displayed a stronger surface adhesion rate upon induction by doxycycline (Fig. 8, lower panels, *PGA59*, + Dox). Both frequency of cell surface-tip adhesion events (82%) and adhesion forces (ranging from 1 up to 5 nN) were higher than those observed for *PGA22* overexpression (Fig. 8, lower panels). Taken together, our AFM analyses indicated that *PGA22* and *PGA59* overexpression imparted significant adhesion forces to single *C. albicans* cells.

### Overexpression of *PGA22* alters the structure of the *C. albicans* cell wall

**Figure 9:**
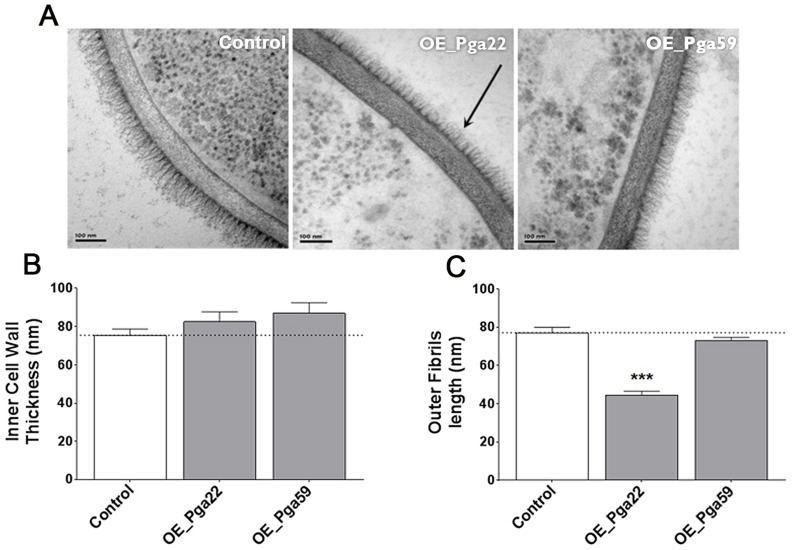
Transmission electron microscopy of the cell wall upon overexpression of the *PGA22* and *PGA59* genes. Overexpression strains for *PGA22* and *PGA59* and the SC5314 control strain were grown overnight in YPD in the presence or absence of 50 µg.mL^−1^ doxycycline and the structure of their cell wall analyzed by transmission electron microscopy. Representative images of the cell wall of the three strains are shown in (A) and allow visualization of the cell wall inner layer containing the skeletal polysaccharides chitin, β-1,3-gucan and β-1,6-gucan, and the cell wall fibrillar outer layer enriched with *O-*linked and *N-*linked mannose polymers (mannans) covalently associated with GPI-anchored proteins attached to the β-1,3-gucan skeleton. The black arrow highlights the reduced outer layer in the *PGA22-*overexpression strain. (B) Inner cell wall thickness and (C) Outer fibrils length; Data are average measurements taken for 30 individual cells of each strain and standard error of means are shown. Significant results according to Student's *t-*tests are represented on the top of each bar (asterisk); *** p≤0.001.

The *C. albicans* cell wall is characterized by an inner layer containing the skeletal polysaccharides chitin, β-1,3-glucan and β-1,6-glucan, and a fibrillar outer layer enriched with *O-*linked and *N-*linked mannose polymers (mannans) covalently associated with proteins. The major class of cell wall proteins are GPI-modified proteins attached to the β-1,3-glucan skeleton by β-1,6 linkages [41], [42]. We reasoned that overexpressing predicted GPI-anchored proteins could result in an abnormal cell wall structure, leading to modified adherence to the substrate and/or other cells. The cell wall architecture of strains overexpressing *PGA22* and *PGA59* were analyzed using transmission electron microscopy (TEM). Results presented in Fig. 9 showed that the *PGA22*-overexpressing strain displayed a thinner outer fibrillar layer as compared to the control strain and the *PGA59-*overexpressing strain. In contrast, the inner cell wall thickness of these strains was similar (Fig. 9B). Alterations in the cell wall structure of the *PGA22*-overexpression mutant may contribute to its modified ability to bind Thermanox and/or aggregate.

### Genome-wide transcript profiling data correlate with perturbation of the *C. albicans* cell wall upon *PGA22* overexpression

To better understand how *PGA22* overexpression affects the cell wall structure, we additionally performed transcript profiling of the *PGA22*-overexpression strain under the same growth conditions used for the TEM analysis (Fig. 9). Three independently grown *PGA22*-overexpression strains were treated or not with 50 µg.mL^−1^ doxycycline for 16 h followed by total RNA extraction, reverse transcription, labeling and hybridization to a custom-designed *C. albicans* ORF microarray that was described previously [36]. Analysis of the control strain (see below) indicated that doxycycline treatment did not affect gene expression under these conditions (S10 Figure). Using a fold-change cut-off of 1.5 and a p-value threshold of ≤0.05, 37 genes were upregulated upon *PGA22* overexpression (Table 3; see Materials and Methods for details and S5 Table for complete transcript profiling data). A significant proportion of cell wall related genes were among the induced genes, including genes encoding (or predicted to encode) GPI-anchored proteins (*PGA29*/*RHD3*, *CRH11*), a chitinase (*CHT3*) and a chitin synthase (*CHS1*), cell wall proteins (*RBE1, SCW1*) as well as putative adhesins (*PGA35*/*FGR41*, *PGA38* and *ORF19.5267*) (Table 3). Gene Ontology term enrichment analysis revealed a strong overrepresentation of the term “Cell wall” (p = 0.001) among the transcriptionally induced genes. On the other hand, although 100 genes were significantly downregulated (fold-change <−1.5; p-value <0.05; S5 Table), no significant gene ontology enrichment was found. However, we noticed the presence of genes encoding mannosyltransferases (*MNN12*, *MNT1*, *KTR4*, *RHD1*) and genes linked to or affecting mannosyltransferase activity (*VRG4*, *SMF12*, *SKN1*) in the set of downregulated genes (S5 Table, See discussion). We confirmed the expression microarray data by RT-qPCR analyses of selected targets (S10 Figure), using the parental BWP17 strain carrying the empty vector (BWP17AH-CIp10-P*_TET_*-GTW, S4 Table) as a negative control for doxycycline-inducible expression (Control, S10 Figure). Taken together, our transcript profiling data suggested that *PGA22* overexpression leads to perturbation of the expression of cell wall genes, consistent with a role of *PGA22* in *C. albicans* cell wall structure and/or function.

**Table 3:** List of genes whose expression was significantly upregulated upon PGA22 overexpression.

orf19^a^	Gene name^b^	Description^c^	Fold Change^d^
*ORF19.3738*	*PGA22*	Putative GPI-anchored protein; adhesin-like protein	13.6*
*ORF19.1670*	*BRO1*	Class E vacuolar protein sorting factor; role in transport from multivesicular body to vacuole	2.2
*ORF19.5267*		Putative cell wall adhesin-like protein	2.2*
*ORF19.344*		Unknown function	2.1*
*ORF19.4910*	*FGR41*	Putative GPI-anchored adhesin-like protein	1.9*
*ORF19.7218*	*RBE1*	Pry family cell wall protein	1.9*
*ORF19.7586*	*CHT3*	Major chitinase	1.8*
*ORF19.1212*		Unknown function	1.8
*ORF19.5305*	*RHD3*	GPI-anchored yeast-associated cell wall protein	1.7
*ORF19.3893*	*SCW11*	Cell wall protein	1.7
*ORF19.6010*	*CDC5*	Polo-like kinase; member of conserved Mcm1 regulon	1.7*
*ORF19.5188*	*CHS1*	Chitin synthase; essential; for primary septum synthesis in yeast and hyphae	1.7
*ORF19.4959*		Unknown function	1.6
*ORF19.1538*	*TLG2*	Putative syntaxin-like t-SNARE	1.6
*ORF19.5343*	*ASH1*	GATA-like transcription factor; localizes to daughter cell, hyphal tip cell nuclei	1.6*
*ORF19.2706*	*CRH11*	GPI-anchored cell wall transglycosylase	1.6
*ORF19.6393*		Putative Arf3p GTPase activating protein	1.6*
*ORF19.5402*		Unknown function	1.6
*ORF19.5805*	*DLD1*	Putative D-lactate dehydrogenase	1.6
*ORF19.4645*	*BEM1*	Protein required for wild-type budding, hyphal growth, and virulence in a mouse systemic infection	1.6*
*ORF19.639*		Unknown function	1.6
*ORF19.3606*		Ortholog of *S. cereviae* Sna4 vacuolar outer membrane protein	1.6
*ORF19.3573*	*PEX6*	Ortholog(s) have ATPase activity	1.6
*ORF19.4623*		Unknown function	1.6
*ORF19.144*	*SNU114*	Protein similar to *S. cerevisiae* Snu114p, which is an RNA helicase involved in pre-mRNA splicing	1.6
*ORF19.2758*	*PGA38*	Putative adhesin-like GPI-anchored protein repressed during cell wall regeneration	1.5
*ORF19.5611*		Predicted 3-methylbutanol: NADP oxidoreductase and methylglyoxal reductase	1.5
*ORF19.1146*		Unknown function	1.5
*ORF19.1542*	*HEX3*	Protein similar to *S. cerevisiae* Hex3p involved in DNA damage response	1.5
*ORF19.5238*		Unknown function	1.5
*ORF19.6209*		Predicted membrane transporter	1.5
*ORF19.539*	*LAP3*	Putative aminopeptidase	1.5
*ORF19.1446*	*CLB2*	B-type mitotic cyclin	1.5
*ORF19.2387*		Putative tRNA-Pro synthetase	1.5
*ORF19.2791*	*BBC1*	Putative SH3-domain-containing protein	1.5
*ORF19.4897*	*SFH5*	Putative phosphatidylinositol transporter	1.5
*ORF19.6102*	*RCA1*	bZIP domain-containing transcription factor of the ATF/CREB family involved in regulation of carbonic anhydrases; controls CO_2_ sensing	1.5*
*ORF19.7277*		Predicted ORF in retrotransposon Zorro2 with similarity to zinc finger-containing retroviral nucleocapsid proteins	1.5

a orf19 nomenclature as described in Assembly 21 of the *Candida albicans* genome (see the *Candida* genome database at www.candidagenome.org)

b Gene name according to CGD (www.candidagenome.org)

c Short description of protein function

d Fold-change (≥1.5) in microarray data with p-values ≤0.05 (see Materials and Methods). *Asterisks denote a fold-change value resulting from the average of two independent probes of the same ORF (see Materials and Methods for details).

### Expression of *C. albicans PGA22* induces cell cluster formation and affects cell wall structure in a heterologous context

**Figure 10:**
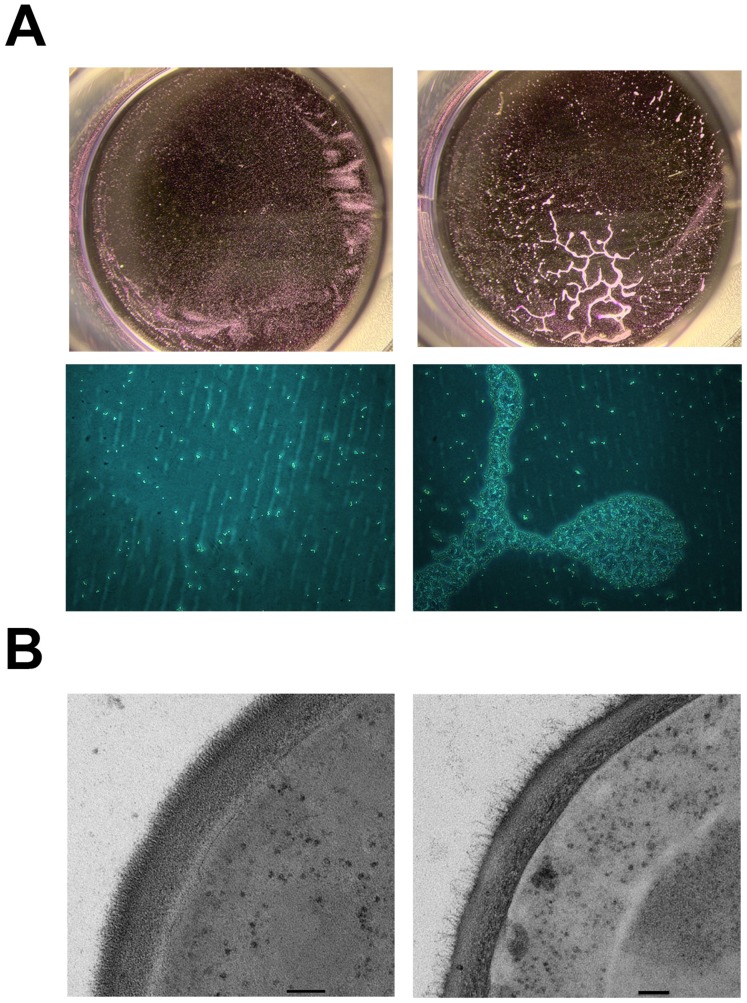
Constitutive expression of *PGA22* in *S. cerevisiae* triggers cell clustering and alters cell wall structure. (A) *S. cerevisiae* BY4742 constitutively expressing *C. albicans PGA22* (right panels) was tested for adhesion to a 24-well plate and compared to a control strain (left panels); after crystal violet treatment and rinsing, cells adhered to the wells were photographed using a stereomicroscope (upper panels) or an inverted microscope (20x magnification, bottom panels). (B) Transmission electron microscopy was performed on *S. cerevisiae* constitutively expressing *PGA22* (right panel) as well as the control (left panel). The cell wall structure of the *S. cerevisiae* strain expressing *PGA22* is disturbed; the outer fibrillar layer is less dense than the control. Scale bars: 100 nm.

To test whether the *Candida*-specific *PGA22* gene may confer cell-to-cell adhesion or an aggregation phenotype in a heterologous context, we expressed *PGA22* in the non-adherent yeast *S. cerevisiae* using a surface display system [43]. Briefly, a version of *PGA22* deleted for the predicted GPI anchor signal was fused to a *S. cerevisiae* cell wall targeting signal, and constitutively expressed from the *TEF1* promoter. *S. cerevisiae* cells expressing this fusion protein were allowed to adhere to a 24-well polystyrene plate for 1 h, and the biomass was measured with crystal violet staining, after thorough rinsing of the wells. The *S. cerevisiae* strain transformed with the empty vector was used as a negative control. There was no significant difference in the biomass between the two strains, but examination of the polystyrene surfaces after rinsing showed aggregation of the *PGA22*-expressing *S. cerevisiae* strain (Fig. 10A). The cell wall structures of the control and *PGA22*-expressing strains were then analyzed by TEM. When expressing *PGA22*, *S. cerevisiae* exhibited a cell wall with a less dense fibrillar outer layer, reminiscent of the structure observed when overexpressing *PGA22* in *C. albicans* (Fig. 9A and 10B). Thus, excess of *PGA22* seemed to cause major structural modifications of the outer cell wall, which may modify cell-to-cell and cell-to-surface adhesion properties.

### Inactivation of *PGA22* also impacts adherence, biofilm formation and cell wall structure

**Figure 11:**
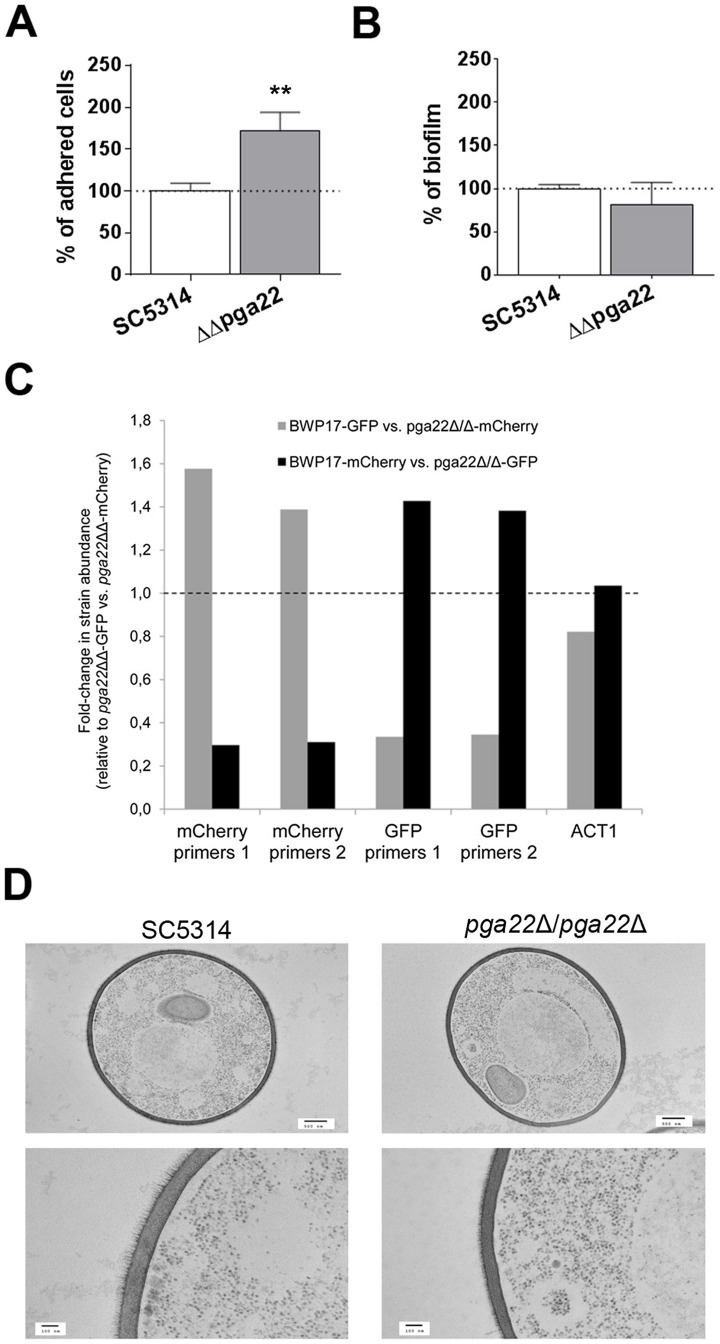
*PGA22* inactivation results in increased adherence, higher occupancy of a mixed biofilm and alteration of the cell wall outer layer. (A) Adherence of the *pga22*Δ/*pga22*Δ mutant (ΔΔ*pga22*) and the SC5314 control strain to Thermanox was quantified. Data were normalized using as reference the adherence shown by the control strain SC5314. Significance of the adherence differences relative to the control strain adherence were assessed using Student's t-tests performed on at least 15 pictures for each strain and results are represented on top of the bar (** p≤0.01). (B) Biofilms of the ΔΔ*pga22* and SC5314 strains were developed in a microfermentor under a continuous flow of GHAUM, and their dry weight was measured after 40 h. The dry mass of the biofilms is expressed in percent of the average dry mass of biofilms formed by the control strain SC5314. Data are average of at least 3 replicates and standard errors of means are shown. No significant difference was observed with a Student's t-test. (C) Biofilms were developed for 40 h using as an inoculum a 1∶1 mixture of either a GFP- or mCherry-expressing parental strain (BWP17-GFP or BWP17-mCherry, S4 Table) and a mCherry- or GFP-expressing *pga22*Δ/*pga22*Δ mutant (ΔΔ*pga22*-mCherry or ΔΔ*pga22*-GFP, S4 Table), respectively. As a control, biofilm growth of a 1∶1 mixture of ΔΔ*pga22*-mCherry and ΔΔ*pga22*-GFP was used. The abundance of mCherry or GFP relative to their respective quantification in the 1∶1 mixture of ΔΔ*pga22*-GFP and ΔΔ*pga22*-mCherry (control) was quantified by qPCR using two independent sets of primers for the mCherry (mCherry primers 1 and mCherry primers 2) and GFP (GFP primers 1 and GFP primers 2) genes. Data were averaged for two biological replicates. (D) Transmission electron microscopy of strains ΔΔ*pga22* and SC5314. Cell structure of the indicated strain is shown in the upper panels (scale bar: 500 nm). Ultrastructure of the cell wall is shown in the lower panel (scale bar: 100 nm). Representative images are shown and allow visualization of the cell wall inner and outer layers. A reduction in the thickness of the outer layer is observed in the ΔΔ*pga22* mutant (right lower panel) as compared to the wild-type strain (left lower panel).

In order to get further insight on the role of *PGA22*, we investigated the behavior of a *pga22*Δ*/pga22*Δ strain upon adherence to and biofilm formation on Thermanox using an existing deletion mutant (S4 Table). We found that the *pga22*Δ*/pga22*Δ strain displayed increased adherence to Thermanox (Fig. 11A), although it was not significantly altered for biofilm formation in the microfermentor system (Fig. 11B). We also tested the effect of deleting *PGA22* on competitive biofilm growth (Fig. 11C). We generated *pga22*Δ/*pga22*Δ mutants expressing either GFP or mCherry (ΔΔ*pga22*-GFP or ΔΔ*pga22*-mCherry; S4 Table) and mixed each mutant with the parental strain expressing either mCherry or GFP, respectively (BWP17-mCherry or BWP17-GFP; S4 Table), at a 1∶1 ratio, followed by growth for 40 h in the microfermentor to form biofilms. As a control, biofilm growth of a 1∶1 mixture of strains *pga22*Δ/*pga22*Δ expressing GFP and mCherry was used. We quantified the relative strain abundance by qPCR using mCherry and GFP as strain identifiers and two independent primer sets for each gene (Fig. 11C, see Materials and Methods for details). We found that the *pga22*Δ/*pga22*Δ mutant outcompeted the parental BWP17 strain (Fig. 11C), correlating with its increased adherence on Thermanox (Fig. 11A). Finally, TEM revealed that the cell wall of the *pga22*Δ/*pga22*Δ mutant had a thinner outer fibrillar layer as compared to the control strain, while the inner cell wall was unchanged (Fig. 11D). Taken together, these results indicated that lack of *PGA22* resulted in an altered cell wall structure that contributed to increased adherence and occupancy of a multi-strain biofilm and that modifying positively or negatively Pga22 levels in the *C. albicans* cell wall impacted the cell wall structure and function.

## Discussion

We designed a screen to identify *C. albicans* genes that when overexpressed alter planktonic growth fitness or strain abundance in a multi-strain biofilm. Surprisingly, our study showed that over-representation of a *C. albicans* strain in a multi-strain biofilm did not systematically correlate with an increased biomass in single-strain biofilms. This observation reinforces the notion that cell-to-cell interactions play critical roles in the formation of *C. albicans* biofilms.

There was a noteworthy enrichment for cell surface-related genes among the overexpressed genes that conferred increased abundance in the multi-strain biofilm. Indeed, ten genes were included in this category, encoding either predicted GPI-anchored proteins (Ihd1/Pga36, Pga15, Pga19, Pga22, Pga32, Pga37, Pga42, Pga59 and Phr2), or a secreted protein of unknown function (Tos1) with similarity to the predicted GPI-anchored protein Pga52 [42], [44]. Notably, the majority (70%) of these proteins are specific to pathogenic *Candida* species and 5 have orthologs only in *C. dubliniensis* (Pga15, Pga19, Pga32, Pga37, Pga42; [42], [45]). Overexpression of *MSB2* also resulted in increased occupancy of the multi-strain biofilm. Msb2 is a plasma membrane-bound signaling mucin with a heavily glycosylated extracellular domain [46], [47]. Inactivation of *MSB2* leads to a defect in biofilm formation [47], consistent with our observation that its overexpression favors biofilm formation. Relatively little is known about the functions of the nine predicted GPI-anchored proteins, and their localization at the cell membrane or in the cell wall has not been fully investigated. One exception is Phr2, a member of the beta-glucanosyltransferase family, which is covalently attached to the cell wall and has a role in cell wall biogenesis at low pH [48] and no described role in biofilm formation. The three remaining genes in the beta-glucanosyltransferase family, namely *PGA4*/*GAS1*, *PGA5*/*GAS2*, and *PHR3* were not tested in this study. Pga59 is a small, abundant, cell wall GPI-anchored protein whose absence negatively impacts on cell wall integrity and hyphal morphogenesis [49]. While the *PGA59* gene is highly expressed in biofilms [49], [50], it is not strictly required for biofilm formation under the conditions analyzed to date [49]. Overexpression of *PGA62*, a paralog of *PGA59*, also increased *C. albicans* occupancy of the multi-strain biofilm but to a lower extent than *PGA59* overexpression (S3 Table). *PGA15, PGA22, PGA37* and *PGA42* are members of the Cell Surface-Targets of Adherence Regulators (CSTAR) group of genes [25]. *PGA15, PGA41* and *PGA42* are members of a *C. albicans*-specific gene family [44]. Overexpression of *PGA41* also showed a tendency for increased *C. albicans* occupancy of the multi-strain biofilm (S3 Table). Overexpression of the different members of a gene family did not always result in similar phenotypes in the ST-OE screen. For instance, *PGA37* and *PGA57* are paralogs but only overexpression of *PGA37* resulted in increased occupancy of the multi-strain biofilm under the conditions tested (S3 Table). We found that many of the genes identified in our screen were upregulated at/in different steps and/or models of biofilm development, including *PHR2* [51], *TOS1* [34], [52], *MSB2*, *PGA32*, *PGA22*, *BEM2*, *IHD1* and *PGA37* [34]. For instance, *PHR2* appears induced during late steps of biofilm development (*i*.*e*. mature biofilms) [51], whereas *TOS1* appears induced during early events [52].

The behavior of the overexpression strains for these ten cell surface-related genes in single-strain biofilms did not follow a general pattern. Overexpression of some genes (*eg PGA59* and *IHD1*) resulted in increased adherence and an increased or unchanged biofilm biomass. In contrast, overexpression of other genes had a negative impact on adherence and no impact on biofilm formation (*PGA19*, *PGA32* and *PGA37*), a positive impact on adherence and a negative impact on biofilm formation (*PGA15, PGA22*) or no discernible impact (*PGA42*, *PHR2* and *TOS1*). Taken together, these results suggest that the ability of *C. albicans* strains to adhere to the biofilm substratum is not the only defining component of biofilm development. In addition, the discrepancies observed when testing single-strain biofilm formation and strain occupancy in a multi-strain biofilm indicate that the environment provided by a multi-strain biofilm may favor biofilm occupancy by a strain otherwise defective for single-strain biofilm formation. This is illustrated by the *PGA22*-overexpression strain that had increased adherence, but adhered cells were more sensitive to shear forces, due to cell-to-cell clustering, thus delaying the emergence of a single-strain biofilm. Such sensitivity to shear forces was not observed when this strain formed a biofilm in combination with one or several other *C. albicans* strains that expressed *PGA22* at normal levels. Hence the presence of wild type cells may protect the *PGA22-*overexpression strain from the deleterious effects of shear forces. Cooperativeness between strains that express cell surface proteins to different levels has already been observed. Indeed, Nobile *et al.* [15] have shown that *hwp1* and *als1als3* knockout mutants are individually defective in biofilm formation. Yet, the combination of these mutant strains results in the formation of an intact multi-strain biofilm. This suggested that Hwp1 interacts with Als1 and Als3 in order to ensure efficient biofilm formation. Here, we did not identify any functional link between the predicted cell surface proteins studied, but our results indicate that they may participate in cell-to-substrate and cell-to-cell interactions, with different impacts on biofilm formation.

It is currently unknown how *PGA22* precisely confers increased cell-to-cell and cell-to-substrate adhesion. We observed that overexpression of *PGA22* in either *C. albicans* or *S. cerevisiae* had a significant impact on the cell wall structure of both species and caused cell aggregation (Fig. 7 and 10), suggesting that the Pga22 protein might have a more direct effect on the cell wall structure. Our AFM experiments indicated a clustered repartition of the adhesion events on the cell surface of the *C. albicans* cells overexpressing *PGA22*, not seen with *PGA59*. This may indicate the presence of Pga22-enriched cell surface domains and explain the alteration of cell wall structure in both *C. albicans* and *S. cerevisiae*. Our genome-wide transcript profiling data also showed that *PGA22* overexpression triggered alterations in the expression of cell wall genes, including putative adhesins (*e*.*g*. *FGR41*, and *PGA38*) or chitin synthesis and remodeling genes (*CHS1* and *CHT3*) that may participate in conferring the adhesion phenotype or be a consequence of altering the cell wall structure and/or function by Pga22 overproduction (Table 3, S5 Table, S10 Figure). *PGA22* overexpression also correlated with downregulation of mannosyltransferase-encoding genes (*MNN12*, *MNT1*, *KTR4* and *RHD1*, S5 Table) as well as genes associated with mannosyltransferase activity and wall maintenance (*VRG4*, *SMF12*, *SKN1*, S5 Table), which may impact on the production of mannoproteins at the external layer of the cell wall as well as affect the overall wall structure and composition. Consistently, TEM analyses revealed that the *PGA22*-overexpressing strain had an altered mannoprotein-rich outer fibrillar layer (Fig. 9). Our preliminary results also showed that overexpression of *PGA22* reduced the cell wall protein content and decreased Concanavalin A staining, indicative of reduced mannoprotein abundance. On the other hand, both overexpression and deletion of *PGA22* cause increased adherence and increased biofilm formation under competitive growth with a wild-type strain (Fig. 4 and S7 Figure). It is possible that cells compensate for *PGA22* absence by producing other adhesion proteins. Previous studies have reported cases where a gene deletion phenocopied the overexpresser. For instance both deletion and overexpression of α-1,2-mannosyltransferases similarly altered cell wall integrity in *Mycobacterium smegmatis* and *Mycobacterium tuberculosis* [53]. Both overexpression and deletion of *SFL1*, encoding a transcription factor that controls the yeast-to-hyphae transition, attenuated virulence of *C. albicans* in a mouse model of systemic infection [54]. Clearly, more studies are needed for a better understanding of the complexity of *PGA22* function during *C. albicans* biofilm development. Further experiments will also be needed in order to understand the basis for the changes in adherence and biofilm formation of overexpression strains for other cell wall genes identified in this study. These changes may reflect subtle modifications in the physical properties of their cell walls, including altered charge and hydrophobicity and compensatory changes in the cell wall proteome, as well as modification of the extracellular matrix.

Our overexpression screen uses the conditional overexpression system pNIM1 [30], [62] that has the advantage of inducing gene expression under tightly controlled conditions and bypassing the effect of any mutation (e.g. acquired during *C. albicans* transformation) that could interfere with the phenotype. In addition, our validation experiments were performed using independently-generated overexpression strains carrying the alternative doxycycline-inducible overexpression system pNIMX that allows for higher overexpression levels to be achieved [30]. Some limitations of our system include possible doxycycline-dependent effects on the observed phenotypes, even if low concentrations of doxycycline (in the range of 40-50 µg/ml) were used. We provide some data arguing against doxycycline interference with both gene expression (S10 Figure; Control) and phenotype (S9 Figure; Vector-only control). Biofilms are also notorious for their ability to exclude small molecules, such as antifungal agents and antibiotics, and it is possible that doxycycline does not reach deep areas within the mature biofilm. Our biofilm development model relies on a continuous supply of the growth medium with doxycycline, suggesting that at least cells that adhere to or are located within the external surface of the biofilm are constantly exposed to doxycycline. Furthermore, strains overproducing Pga22 express high levels of *PGA22* even after 16 h of exposure to doxycycline (S10 Figure) indicating that doxycycline promoter-driven transcripts are sustained over a long period of time.

Overexpression of several *C. albicans* genes has previously been shown to affect biofilm formation. For instance, overexpression of *ADH5*, *GCA1*, and *GCA2* promoted matrix production, while overexpression of *CSH1* and *IFD6* inhibited it [23] and *PES1* overexpression increased the dispersal step [55]. These genes were not included in our overexpression collection. Other genes, with known biofilm phenotypes when overexpressed, were included in our collection e.g. *NRG1, UME6* and *GAT2/BRG1.* Overexpression of *NRG1* repressed morphogenesis and resulted in the formation of yeast-only biofilms [56]. Overexpression of *UME6* and *GAT2/BRG1* triggered hyphal formation independently of the presence of hypha-inducing cues [32] and resulted in the formation of hyperfilamentous biofilms and increased biofilm biomass, respectively [32], [56], [57]. Despite these previous findings the *NRG1*-, *UME6-* and *GAT2/BRG1-*overexpression strains did not display altered occupancy in our multi-strain biofilm model. This reinforces the notion that the behavior of individual strains in a multi-strain biofilm cannot be directly predicted from their behavior in a single-strain biofilm model. In fact, the majority of the genes identified through the ST-OE biofilm screen did not have an impact on morphogenesis when overexpressed (S2B Figure), although this process is of central importance to biofilm formation [8]. Our unpublished data showed that only overexpression of *ORF19.3459/MCK1* resulted in a filamentation phenotype at 37°C (but not 30°C) in media that do not normally promote hyphal growth. Thus, this strain might show normal adherence but increased hyphal growth and representation in a biofilm. In addition, overexpression of two cell polarity genes *CDC24* and *BEM2* [58] resulted in increased representation in the multi-strain biofilm although the underlying mechanism remains to be investigated.

We found that overexpression of four genes involved in the regulation of cell-cycle progression and the DNA-damage response, namely *RAD53*, *RAD51*, *PIN4* and *ORF19.2781*, and one gene known for its role in the regulation of *C. albicans* morphogenesis (*SFL2*), resulted in decreased fitness upon planktonic growth with doubling times increasing by 3-27% when strains were grown individually (Fig. 2B). Our results are consistent with the *S. cerevisiae* phenotypes associated with overexpression of the orthologs of these five genes [27], [59]. One observation that emerges from our study is the low number of *C. albicans* genes that when overexpressed resulted in decreased fitness (5/531; 1%). This contrasts with the observation of Douglas *et al.* [59] who used a similar setting (growth of a pool of ∼5,100 barcoded P*_GAL4_*-dependent *S. cerevisiae* overexpression strains) and identified 361 (7.1%) strains with decreased fitness after 20 generations under inducing conditions. This may reflect a weaker level of overexpression from the P*_TET_* promoter used in our study. Indeed, when using a modified transactivation system that induces higher expression from the P*_TET_* promoter (pNIMX, [30]) and a collection of 257 overexpression strains for genes largely overlapping those analyzed here, 18 strains (7.0%) were identified with a fitness defect, including those overexpressing *RAD53* and *SFL2* [30]. A second interesting observation is the lack of genes whose overexpression conferred increased fitness. This was also the case when higher levels of expression were driven from the pNIMX system (S. Znaidi and C. d'Enfert, unpublished) and in the study of ∼5,100 *S. cerevisiae* overexpression strains [59]. It is likely that use of a relatively rich medium provided optimal growth conditions and therefore limited discriminatory capacity for increased fitness. Our approach also identified four genes, namely *ASH1, ORF19.2781, PRR2* and *STB5* whose overexpression led through unexplored mechanisms to under-representation of the corresponding strains in a multi-strain biofilm (Fig. 3, Table 1). It is notable that among these genes, only *ORF19.2781* resulted in a lower fitness when overexpressed in planktonic culture, suggesting that fitness determinants may differ depending on growth conditions.

In conclusion, our results illustrate the power of using signature tagging in conjunction with gene overexpression for the identification of genes involved in biofilm formation and more general processes pertaining to *C. albicans* virulence. This warrants our current development of a genome-wide collection of *C. albicans* overexpression strains [60]. Moreover, our results reveal how targeted changes in the cell wall proteome differentially alter *C. albicans* ability to form single- and multi-strain biofilms, re-emphasizing the importance of the cell wall and cell-cell interactions in *C. albicans* pathobiology.

## Materials and Methods

### *C. albicans* strains

*C. albicans* overexpression strains used in this study have been derived from 294 barcoded integrative overexpression plasmids described previously [30], as well as 237 novel barcoded plasmids for overexpression of transcription factors and signaling components (90), cell wall-related proteins (61) and genes involved in DNA replication, recombination and repair (86; see S1 Table for a list of all ORFs included, primers used for their amplification and corresponding barcodes). Briefly, for this latter set of 237 plasmids, the respective ORFs were PCR amplified using chimeric primers followed by recombination-mediated transfer into the Gateway donor vector pDONR207 [30], [61]. The set of pDONR207 derivatives was fully sequenced to ascertain that no unintended mutations were introduced during PCR amplification. The pDONR207-ORF plasmids were then used in a Gateway LR reaction together with barcoded derivatives of the CIp10-P*_TET_*-GTW vector [30], carrying a *TET* promoter (P*_TET_*, [62]). All barcoded overexpression vectors were linearized with *Stu*I and used to transform either CEC1121, a derivative of SN148 [63] (S4 Table), or CEC1429, a derivative of CAI4 [64] (S4 Table), both strains harbor the pNIM1 plasmid [62] for doxycycline-regulated expression from the P*_TET_* promoter. Transformants were selected and checked as described yielding 531 overexpression strains [61]. Selected overexpression plasmids were also used to transform strains CEC3783 or CEC3781 carrying the pNIMX plasmid (S4 Table; [30]) for doxycycline-regulated expression from the P*_TET_* promoter and either a P*_TDH3_-*BFP or P*_TDH3_-*GFP gene fusion for constitutive expression of BFP or GFP, respectively. Constructs with GFP and BFP were integrated between *PGA59* and *PGA62*. *P_TET_* was induced with 50 µg.mL^−1^ of doxycycline in all experiments.

The *C. albicans *ΔΔ*pga22* loss-of-function mutant was generated in BWP17 [65] by successive replacement of the complete ORF in the two alleles using PCR-generated disruption cassettes flanked by 100 bp of target homology region as previously described [66]. The disruption cassettes were amplified using oligonucleotides 3738J5DR and 3738J3DR described in S6 Table and *ARG4-* or *HIS1-*bearing plasmids. The resulting transformants were verified by PCR and one clone was selected for subsequent transformation with plasmid CIp10 [67] yielding the prototrophic ΔΔ*pga22* mutant (S4 Table). Alternatively, the selected clone was transformed with CIp10 derivatives harboring either the GFP gene placed under the control of the *C. albicans TDH3* promoter or the mCherry gene placed under the control of the *C. albicans ADH1* promoter, yielding strains ΔΔ*pga22-*GFP and ΔΔ*pga22-*mCherry, respectively.

### Preparation of strain pools

The 531 signature-tagged overexpression strains were thawed on Nunc omnitray plates (Thermo Scientific) containing YPD (1% Yeast Extract, 2% Bacto-Peptone, 2% D-glucose)-agar using a 96 pin replicator and allowed to grow for 6 days at 30°C. No significant colony size alterations were recorded. 10 mL of YPD were added to each plate and colonies were scraped off using a cell spreader. Strains were pooled in ∼100 mL YPD/15% glycerol at a concentration of ∼57 OD_600_ (optical density at 600 nm) units.mL^−1^, aliquoted in 2-mL tubes and frozen at −80°C.

### Planktonic fitness assay

The overexpression strain pool was grown at 30°C with agitation (200 rpm) for 16 generations in GHAUM medium, a synthetic defined medium (0.67% Yeast Nitrogen Base, 2% D-glucose) supplemented with histidine, arginine, uridine and methionine (at final concentrations of 1 mg.mL^−1^, 1 mg.mL^−1^, 0.02 mg.mL^−1^ and 2 mg.mL^−1^, respectively), in the absence or presence of 50 µg.mL^−1^ doxycycline. Genomic DNA was extracted as described for *S. cerevisiae* in Rose *et al.* [68] from strain pools, followed by PCR-amplification of the barcodes using primers CipSAC2-UP-2 and CipSAC2-DWN-2 (3 min at 94°C; followed by 35 cycles of 30 sec at 94°C, 30 sec at 50°C, and 30 sec at 72°C; and a final step of 7 min at 72°C) (see S6 Table for primers used in this study). The PCR products were then subjected to indirect differential fluorescent dye labeling (Cy5 for Dox-treated, Cy3 for untreated pools). Labeled DNA was resuspended in 50 µL DigEasy Hyb solution (Roche), incubated at 95°C for 5 min, snap-cooled on ice and directly deposited on a barcode microarray (Agilent Technologies, GEO platform # GPL17420) containing: i) ∼12 on-chip replicates of both sense and antisense DNA sequences complementary to 657 tags (representing 531 strain tags +126 unused tags) and ii) different negative control spots (Agilent reference). Hybridization was performed overnight at 25°C, followed by washing and scanning of the arrays using GenePix 4200 AL scanner (Molecular Devices). This experiment was repeated twice independently. Microarray data were analyzed using two distinct data processing softwares: GeneSpring GX 11 (Agilent Technologies) and ArrayPipe v2.0 [69]. Z-score (i.e. number of standard deviations from the population mean) calculations were performed using ArrayPipe v2.0. The thresholds for GeneSpring were kept at Fold Change values equal or superior to 2 and p-values equal or inferior to 0.05, while thresholds for ArrayPipe v2.0 were absolute Z-score values equal or above 1.5 and p-values equal or below 0.05. Only strains that met both algorithm thresholds for both sense and antisense barcode fluorescence signals were kept as altering planktonic growth. Microarray data have been deposited at GEO under accession number GSE48647 and Z-score and fold-change data are available in S2 Table.

### Confirmation of the microarray fitness data by liquid growth assay

Strains were individually grown three-times independently in 96-well plates at a starting optical density (OD_600_) of 0.1 in 100 µL of YPD supplemented with or without 50 µg.mL^−1^ doxycycline. The OD_600_ was measured every 5 min using a Tecan Infinite 200 reader. Tecan OD_600_ readings were converted into “flask OD_600_” reading using the following formula: OD_Flask_  =  OD_Tecan_ ×12.2716–1.0543 [70] and doubling times were calculated within the exponential growth interval as previously described [71].

### Biofilm formation in a continuous-flow fermentor system

The inoculum was prepared from an early-stationary-phase culture of either the pool of overexpression strains, a combination of two equally represented strains or individual strains grown in flasks at 30°C in an orbital shaker. Cells were grown in GHAUM medium with or without 50 µg.mL^−1^ doxycycline, each inoculum was then diluted to an OD_600_ of 1 in fresh GHAUM medium with or without 50 µg.mL^−1^ doxycycline and left at room temperature for 30 min, to allow further overexpression. Plastic slides (Thermanox; Nunc) were immersed in the inoculum for 30 min at room temperature to allow adherence of cells to the plastic substrate. The plastic slides were then transferred to the glass vessel of a 40-mL incubation chamber [50]. This vessel has two glass tubes inserted to drive the entry of medium and air, while used medium is evacuated through a third tube. The flow of GHAUM medium is controlled by a recirculation pump (Ismatec) set at 0.6 mL.min^−1^ and pushed by pressured air supplied at 10^5^ Pa, conditions minimizing planktonic phase growth and promoting biofilm formation. The chambers with the plastic substrate were incubated at 37°C and biofilms (8 independent biological replicates) were grown for 40 h followed by genomic DNA extraction, barcode amplification and differential labeling (Dox-treated samples with Cy5, untreated samples with Cy3) and hybridization to barcode microarrays as described above. We performed two independent analyses of our microarray data using the Arraypipe [69] or GeneSpring softwares. Arraypipe analyses identified 29 genes with absolute Z-score values above or equal to 1.5, fold change above or equal to 2, and p value below or equal to 0.05 (Fig. 3, Table 1, S3 Table), while GeneSpring analyses identified 21 genes when the last two selection criteria described above were used (Table 1 and S3 Table). Microarray data have been deposited at GEO under accession number GSE48647.

### Quantification of strains in mixed biofilms using quantitative PCR

Mixed biofilms with two strains expressing either the BFP or the GFP genes under the control of the P*_TDH3_* promoter were grown for 40 h as described above. Plastic substrates were then recovered and immersed in 25 mL of PBS. Biofilms were detached from the plastic substrates by vortexing twice for 15 sec, and collected by centrifugation. Genomic DNA was extracted using MasterPure Yeast DNA Purification Kit (Epicentre), and quantified using a NanoVue Plus (GEHealthcare Life Sciences); all samples were adjusted to a DNA concentration of 100 ng.µL^−1^. 10 ng.µL^−1^ and 1 ng.µL^−1^ dilutions were used as templates for quantitative PCR (qPCR), with the following protocol: 0.2 µM of each primer was added to SYBR Green (Invitrogen) and 5 µL of DNA, in a total reaction volume of 25 µL; qPCR was performed as follow: 3 min at 94°C, followed by 35 cycles of 30 sec at 94°C, 30 sec at 58°C, and 30 sec at 72°C, and a final step of 7 min at 72°C. Each sample was tested for amplification within the BFP and GFP coding regions (using the primers BFPpFwd and BFPpRev, and GFPpFwd and GFPpRev respectively; S6 Table). The resulting Ct value of each amplification was analyzed in order to assess the ratio between the GFP- and BFP-strains within the biofilm, in the induced (with Dox) and non-induced (without Dox) samples. Six replicates for each strain were analyzed through a Student's *t*-test. For competitive growth of the *ΔΔpga22* mutant versus the parental BWP17 strain, two independent sets of primers for mCherry (primers 1, mCherry2-FWD and mCherry2-REV; primers 2, mCherry-FWD and mCherry-REV) and GFP (primers 1, GFPpFwd and GFPpRev; primers 2, GFP.RT.fw and GFP.RT.rv) were used (S6 Table) and two independent experiments were averaged. The *TEF3* gene was used as a calibrator and the *ACT1* gene was used as a control (primers ACT1-FWD and ACT1-REV, S6 Table).

### Biofilm dry weight measurement

Biofilms were grown and recovered from the substrate after 18, 24, 40, 48 and 65 h of growth as described above. The PBS solution containing the detached biofilm was vacuum-filtered through a 1.2 µm filter (Millipore); the filter was dried at 60–65°C for 2–3 days and then weighed on a precision scale (Mettler AE200; Mettler Toledo) to obtain the dry mass of the biofilm. A minimum of 3 replicates were analyzed through a Student's *t-*test.

### Adherence

After adherence as described above, the plastic substrates were washed three times in PBS to remove non-adherent cells, and mounted on a glass slide for observation with a Leica DM RXA microscope, using an objective at 10× magnification or an oil-immersed objective at 40× magnification. Pictures of the substrates were taken and 15 fields were counted for each strain per condition (presence or absence of doxycycline), except for the strains overexpressing *PGA42*, *TOS1* and *PHR2*, with 10 fields each. The replicate measurements were analyzed through a Student's *t-*test. The same test was performed for the ΔΔ*pga22* knockout strain, without doxycycline, and the results were compared to the wild-type strain SC5314. Pictures of the substrates were taken and 20 fields were counted for each strain.

### Atomic Force Microscopy (AFM) analysis of cell surface adhesion

10 mL of YPD (1% Yeast Extract, 2% Bacto-Peptone, 2% D-glucose) liquid medium were inoculated with *PGA22*-overexpression strain, *PGA59-*overexpression strain, and the control strain with empty plasmid (CEC3785), and incubated ON at 30°C, in an orbital shaker (180 rpm). A 10 µL aliquot of each culture was then diluted to 10 mL in fresh YPD medium with or without 50 µg.mL^−1^ doxycycline, and allowed to grow for 16 hours to allow overexpression of the targeted protein. 5 mL of the cell culture were then quickly centrifuged, washed with 5 mL of acetate buffer (18 mM CH_3_COONa, 1 mM CaCl_2_ and 1 mM MnCl_2_, pH 5.2) and resuspended in 3 mL of the same buffer. 100 µL of this cell suspension were deposited on a freshly oxygen-activated microstructured PolyDiMethylSiloxane (PDMS) stamp. Cells were immobilized in the PDMS stamps as described elsewhere [72], and immersed in the same acetate buffer. To get statistical significance of the AFM data, about 10–15 cells have been analyzed from three independent experiments for each strain. AFM experiments were conducted on a Nanowizard III from JPK Instruments (Berlin, Germany). We used MLCT probes from Bruker probes with a spring constant of 0.02 N.m^−1^ +/−10% measured before each experiment by the thermal noise method. Adhesion force maps were recorded in force volume mode (32×32 or 64×64 force curves). The maximum applied force has been set to 2 nN, the Z displacement to 2 µm and the retract time to 50 ms (with a loading rate of 800,000 pN.s^−1^). Force curves were analyzed using JPK data processing software to extract the maximum adhesion force on each force curve.

### Adherence and washing step in the continuous-flow fermentor system

Following adherence, the plastic substrates were introduced in the continuous-flow fermentor system for 2 h, and then observed under the microscope. 30 fields for each condition (presence or absence of doxycycline) were photographed. The cells that were released and washed away during these 2 h under the continuous-flow conditions were also collected, pelleted and resuspended in 1 mL of PBS, 400 µL of which were counted on a MACS Quant (Mylteni Biotec). Student's *t-*tests were performed.

### Confocal microscopy fluorescence images and quantification

Mixed biofilms with two strains expressing either the BFP or the GFP genes under the control of the P*_TDH3_* promoter were grown for 40 h as described above. Confocal microscopy was then performed on the recovered plastic substrates, using a Zeiss LSM 700 laser scanning confocal microscope on an upright Axio Imager Z2 stand, using a Zeiss W-nACHROPLAN 40X/0.75 working distance 2.1 mm objective; z-stacks of the biofilms were obtained using the blue and green lasers, for the whole biofilm thickness. Z-stacks were then analyzed using Volocity software to acquire the volume occupied by the cells in the green channel (overexpression mutant, expressing GFP) and by the cells in the blue channel (control strain, expressing BFP).

### High-pressure freezing (HPF)-transmission electron microscopy (TEM)

Overexpression strains were grown overnight in YPD in the presence or absence of 50 µg.mL^−1^ doxycycline. *S. cerevisiae* strains were grown overnight at 30°C in liquid YNB N5000 medium (0.17% YNB w/o AA w/o ammonium sulfate; 1% Glc; 0.5% Ammonium sulfate) supplemented with leucine, histidine and lysine at a final concentration of 0.1 mg.mL^−1^. Samples were prepared by high-pressure freezing with an EMPACT2 high-pressure freezer and rapid transport system (Leica Microsystems Ltd., Milton Keynes, United Kingdom). After freezing, cells were freeze-substituted in substitution reagent (1% [wt/vol] OsO4 in acetone) with a Leica EMAFS2. Samples were then embedded in Spurr resin and additional infiltration was provided under a vacuum at 60°C before embedding in Leica FSP specimen containers and polymerizing at 60°C for 48 h. Semithin survey sections, 0.5 µm thick, were stained with 1% toluidine blue to identify areas containing cells. Ultrathin sections (60 nm) were prepared with a Diatome diamond knife on a Leica UC6 ultramicrotome and stained with uranyl acetate and lead citrate for examination with a Philips CM10 transmission microscope (FEI UK Ltd., Cambridge, United Kingdom) and imaging with a Gatan Bioscan 792 (Gatan United Kingdom, Abingdon, United Kingdom). The thicknesses of the inner and outer layers of the cell wall were measured using Image J and by averaging 30 measurements for each cell (*n* = 30 cells). Analyses were performed using Student's *t-*tests.

### Total RNA extraction, expression microarray analyses and qRT-PCR assays

*PGA22* overexpression strain was grown three times independently in YPD medium supplemented or not with 50 µg.mL^−1^ doxycycline during 16 h. Total RNA was extracted using the hot phenol method as described previously [36], followed by first-strand cDNA synthesis and Cy5 (doxycycline-treated samples)/Cy3 (untreated samples) labeling from 20 µg total RNA, using the Superscript III indirect cDNA labeling system (Invitrogen). Purified labeled samples were mixed and hybridized to a *C. albicans* expression array (Agilent Technologies) designed such that two nonoverlapping probe sets target each of 6,105 *C. albicans* ORFs for a total of 15,744 probes, thereby allowing two independent measurements of the mRNA level for a given gene [36]. Hybridization was performed as described elsewhere [36]. Images of Cy5 and Cy3 fluorescence were generated by scanning the expression arrays using an Axon Autoloader 4200AL scanner (Molecular Devices, Downington, PA). Images were subsequently analyzed with the GenePix Pro 6.1.0.2 software (Molecular Devices, Downington, PA). GenePix Results (GPR) files were imported into the Arraypipe 2.0 for spot filtering, background subtraction (limma normexp BG correction) and Lowess global normalization of signal intensities [73]. Replicate arrays (n = 3) were combined and fold-change and P-values (standard Student's *t*-test) were calculated.

For RT-qPCR analyses, the strain CEC3785 (S4 Table) was grown exactly as described above and used as a negative control for doxycycline treatment. Total RNA from both the *PGA22* overexpression- and control strains was extracted using the hot phenol method [36] and reverse transcription (RT) was performed using the SuperScript III first-strand synthesis system using 5 µg of total RNA (Invitrogen, catalog # 18080-051) in a total reaction volume of 20 µL. The qPCR reaction was made of 1 µL from the RT reaction mixture combined with 4 µL of primer mix at 10 pmol.µL^−1^ each (forward and reverse primers of the selected genes, S6 Table), 10 µL of 2X Takyon Rox SYBR MasterMix dTTP Blue (Eurogentec) and 5 µL of H_2_O (total volume  = 20 µL). Q-PCRs were performed in a MicroAmp Optical 96-Well Reaction Plate (Applied Biosystems) using an Eppendorf *realplex^4^* Mastercycler real-time PCR instrument (Eppendorf) with 1 cycle at 50°C for 2 min, 1 cycle at 95°C for 10 min and 50 cycles at 95°C for 15 sec and 58°C for 1 min. Data analysis was performed using the *realplex* software version 2.2 (Eppendorf). For each experiment, threshold cycle (C_T_) values were determined using the *realplex* software. The levels of relative gene expression (n-fold) for the doxycycline-treated samples as compared to the untreated controls of *PGA22*, *ORF19.5267*, *FGR41*, *RBE1*, *CHT3*, *CHS1* and the *ACT1* negative control gene were calculated using the 2^−ΔΔCT^ method, as follows: ΔC_T_  =  C_T_(selected gene) − C_T_(*TEF3* reference gene) and ΔΔC_T_  =  ΔC_T_(doxycycline-treated sample) − ΔCT(untreated control). The *ACT1* gene was used as a negative control. Three independent experiments were performed on different days using 2 biological replicates each time (assumed as n = 6). A two-tailed Student's *t*-test was applied by comparing the doxycycline-treated set to the untreated set. Statistical significance is set as *P*≤0.05.

### Expression of *C. albicans* PGA22 in *S. cerevisiae*

*PGA22* was amplified from SC5314 genomic DNA using PGA22-GTW-fwd and PGA22ΔCter-rev as primers, and recombined into the Gateway donor vector pDONR207 (see above). The resulting plasmid was sequenced, prior to being transferred into *S. cerevisiae* Gateway destination vector pBC542 [43]. Briefly, this centromeric plasmid bears the *TEF* promoter, a Gateway cassette flanked by an inframe HA tag, followed by an inframe *S. cerevisiae* GPI anchor sequence. The pBC542/*CaPGA22* plasmid was used to transform *S. cerevisiae* BY4742, and the resulting strain was tested in an adherence assay as described in Monniot et al [74]. After rinsing, the cells adhered to the 24-well plates were imaged with a Leica M80 stereomicroscope and a Leica DMI6000 inverted microscope, using a HC PLAN APOx20/0.70 objective.

## Supporting Information

S1 Figure **Validation of barcode detection by microarrays.** Genomic DNA was extracted from two independent aliquots of the pooled strains (40 OD units each), followed by PCR-amplification of the barcodes, indirect differential fluorescent dye labeling of the pool-aliquot pair (Cy5 vs. Cy3) and hybridization to barcode microarrays (see materials and methods). (A) Signal scatter plot showing that signal intensities of either the negative control spots (background) or the unused tags were ranging within the log_2_-transformed values of 6–8. This interval also included ∼10% of strain tags, suggesting that the corresponding strains were underrepresented in the pool or that these tags had low hybridization efficiency. (B) Reproducibility of tag detection. The above-described experiment was performed twice independently and Pearson correlation coefficient (R) of the background-corrected raw signals was calculated for each channel. R coefficient values were >0.98, indicating that tag detection was highly reproducible.
(JPG)

S2 Figure **Phenotypic examination of the strains overexpressing the set of genes identified in the competitive overexpression screen during biofilm development.** (A) Microscopic examination (40× magnification) of strains overexpressing *IHD1*/*PGA36*, *PGA15*, *PGA19*, *PGA22*, *PGA32*, *PGA37*, *PGA42*, *PGA59*, *PHR2*, and *TOS1* together with the wild-type control SC5314 during growth in GHAUM liquid medium at 37°C in the presence of doxycycline. (B) Single colonies from the same strains were also grown on solid GHAUM medium at both 30°C and 37°C to test for growth rate alterations.
(JPG)

S3 Figure **Confocal microscopy-acquired fluorescence images of mature biofilms made of GFP-labeled** ***PGA22*** **or** ***PGA59*** **overexpression strains in a 1∶1 mixture with BFP-labeled control parental strain.** (A) Biofilms were developed for 40 h using as an inoculum a 1∶1 mixture of a BFP-expressing control strain and either a GFP-expressing *PGA22-*overexpressing strain (left panels) or a GFP-expressing *PGA59-*overexpressing strain (right panels) in the absence (-Dox, upper panels) or presence (+ Dox, bottom panels) of 50 µg.mL^−1^ doxycycline followed by acquisition of fluorescence intensity images using a confocal microscope. (B) Volume occupied by cells expressing GFP or BFP was quantified using Volocity software, and relative percentage of each strain is represented in the absence (-Dox) or presence (+ Dox) of 50 µg.mL^−1^ doxycycline. Data was averaged for 2 replicates, from two independent experiments, and standard error of means are shown; Student's *t-*tests were performed and results are represented on top of each graph (asterisk); * p≤0.05.
(JPG)

S4 Figure **Higher occupancy of strains overexpressing** ***PGA22*** **and** ***PGA59*** **in a mixed biofilm formed with a wild-type strain.** Biofilms were developed for 40 h using as an inoculum a 1∶1 mixture of a GFP-expressing control strain and either a BFP-expressing *PGA22-*overexpressing strain (A) or a BFP-expressing *PGA59-*overexpressing strain (B) in the absence (-Dox) or presence (+ Dox) of 50 µg.mL^−1^ doxycycline. The abundance of each strain in the mixed biofilm was quantified using qPCR on the GFP and BFP genes. Data averaged for 3 replicates and standard error of means are shown.
(JPG)

S5 Figure **Uninduced overexpression strains for selected cell wall protein genes do not show alteration in adherence to Thermanox.** Adherence of the overexpression strains to Thermanox was quantified following growth in the absence of doxycycline and normalized using the wild-type strain SC5314 as a control. Student's *t-*tests were performed on 10 pictures for each strain and did not reveal significant differences with the wild-type control.
(JPG)

S6 Figure **Overexpression of** ***PGA22*** **and** ***PGA59*** **increases cell adherence to polystyrene substrate.** Adherence of the indicated overexpression strains (OE_Pga22, *PGA22* overexpression; OE_Pga59, *PGA59* overexpression) to polystyrene substrate was quantified following growth of strains in a microtiter plate, during 30 min, in the absence (-Dox) or presence (+ Dox) of 50 µg.mL^−1^ doxycycline. Data were normalized using as a reference the adherence shown by each strain in the absence of overexpression. Statistical tests (Student's *t-*tests) were performed on at least 10 pictures for each strain and results are represented on top of each bar (* p≤0.05).
(JPG)

S7 Figure **Effect of overexpressing** ***PGA22*** **and** ***PGA59*** **on biofilm formation under static growth conditions.** Strains overexpressing *PGA22* (OE_Pga22) or *PGA59* (OE_Pga59) and strain SC5314 (wilde-type control) were individually grown in 6-well plates to form biofilms in the absence (-Dox) or presence (+ Dox) of doxycycline, with one washing step after adherence, followed by another wash after biofilm growth. Biofilms were recovered and dry weight obtained by filtration. Filters were left for 3 days at 65°C and weighed afterwards. Graphs are normalized for the WT strain's biofilm biomass. Data originate from two independent experiments, with at least n = 3 biological replicates (*, *P*≤0.05 from comparison between Pga59-Dox and Pga59+ Dox; ***, *P*≤0.001 from comparison between Pga59+ Dox and SC5314+ Dox using a two-tailed Student's *t*-test).
(JPG)

S8 Figure **Quantification of strain abundance of GFP-labeled** ***PGA22*** **- or** ***PGA59*** **-overexpressing strains relative to BFP-labeled control strain in the lower versus upper layer of mature mixed-strain biofilms.** Biofilms were developed for 40 h using as an inoculum a 1∶1 mixture of a GFP-labeled *PGA22*-overexpressing (A, B) or a GFP-labeled *PGA59*-overexpressing (C, D) strains relative to BFP-labeled control strain in the absence (-Dox) or presence (+ Dox) of 50 µg.mL^−1^ doxycycline followed by quantification of confocal microscopy-acquired GFP and BFP fluorescence signals within the bottom (A, C) and upper (B, D) layers of the mature biofilm (see Materials and Methods for details). *, *P*≤0.05; **, *P*≤0.01 using a two-tailed Student's *t*-test. Data was averaged for 2 replicates, from two independent experiments.
(JPG)

S9 Figure **AFM-derived adhesion measurements in the control strain CEC3785.** Adhesion maps in three independent *C. albicans* cells (Cells #1–3, each analyzed area covers 1×1 µm^2^) recorded on the control strain (CEC3785) with empty vector, treated (+ Dox) or not (-Dox) with 50 µg.mL^−1^ doxycycline during 16 h in YPD. Adhesion scales are shown (Scales; bright yellow, maximum at 1.6 nN; dark red, minimum at 0.0 nN). The corresponding histograms representing the adhesion force repartition (red bars) and representative force curves (grey lines; scales are indicated with light blue bars) are shown at the right of each panel.
(JPG)

S10 Figure **RT-qPCR analysis of selected genes whose expression was induced upon** ***PGA22*** **overexpression.** Quantitative real-time RT-PCR analysis of *PGA22*, *ORF19.5267*, *FGR41*, *RBE1*, *CHT3*, *CHS1* and *ACT1* (as a control). Bars indicate the relative changes in RNA expression of the indicated genes in doxycycline-treated samples versus untreated for the BWP17 parental strain carrying the empty vector (Control, light gray bars) and the derived *PGA22* overexpression strain (black bars). Asterisks denote statistical significance by two-tailed Student's *t*-test (P≤0.05) between the doxycycline-treated and untreated samples. Error bars denote standard deviations. The assay was performed using 3 independent experiments performed on different days, each using two biological replicates (assumed as n = 6 in total).
(JPG)

S1 Table **List of strains included in the overexpression collection.** Headers: **Orf number**, orf19 nomenclature from the Assembly 21 version of the *Candida albicans* genome at the *Candida* Genome Database (CGD, www.candidagenome.org); **Gene name**, gene name according to CGD; **Category**, functional category of the overexpressed open reading frame (ORF); **Recipient strain**, Lab identifier of the strain background carrying the corresponding ORF; **Barcode**, barcode sequence matching the corresponding ORF; **Forward primer**, forward primer used for polymerase chain reaction (PCR)-amplification of the corresponding ORF; **Reverse primer**, reverse primer used for PCR-amplification of the corresponding ORF.
(XLSX)

S2 Table **Complete fitness profiling data of planktonic-cell growth.** Headers: **Probe ID**, Microarray probe identifier; **ORF19 #**, orf19 nomenclature from the Assembly 21 version of the *Candida albicans* genome at the CGD; **Gene name**, gene name according to CGD; **Description**, gene description according to CGD; **fold change**, fold-change abundance of the strain carrying the corresponding ORF treated with doxycycline as compared to untreated control; **Z-score**, Z-score value of the strain carrying the corresponding ORF; **p-student**, p-value of the corresponding fold-change value using a Student's *t*-test (within group).
(XLSX)

S3 Table **Complete fitness profiling data of biofilm growth.** Sheet entitled “Complete biofilm data-Arraypipe” includes data analyzed with Arraypipe (See Materials and Methods). Headers: **Probe ID**, Microarray probe identifier; **ORF19 #**, orf19 nomenclature from the Assembly 21 version of the *Candida albicans* genome at the CGD; **Gene name**, gene name according to CGD; **Description**, gene description according to CGD; **fold change**, fold-change abundance of the strain carrying the corresponding ORF treated with doxycycline as compared to untreated control; **Z-score**, Z-score value of the strain carrying the corresponding ORF; **p-student**, p-value of the corresponding fold-change value using a Student's *t*-test (within group). Sheet entitled “GeneSpring analysis-hits p<0.05” includes the statistically significant data analyzed with GeneSpring using a p-value cut-off of <0.05. Headers: **ID**, Microarray probe identifier; **orf19 #**, orf19 nomenclature from the Assembly 21 version of the *Candida albicans* genome at the CGD; **Gene name**, gene name according to CGD; **Description**, gene description according to CGD; **Fold change**, fold-change abundance of the strain carrying the corresponding ORF treated with doxycycline as compared to untreated control; **p-student (<0.05)**, statistically-significant (p<0.05) p-value of the corresponding fold-change value using a Student's *t*-test (within group).
(XLSX)

S4 Table **Parental and deletion strains used in the course of this work and respective genotypes.** References for the indicated strains are listed at the bottom of S4 Table.
(DOCX)

S5 Table **Complete transcript profiling dataset of** ***PGA22*** **overexpression versus control.** Headers: **Probe ID**, microarray probe identifier for each ORF; ***ORF19***, orf19 nomenclature from the Assembly 21 version of the *Candida albicans* genome at the CGD; **Gene name**, gene name according to CGD; **Description**, gene description according to CGD; **Fold change**, fold-change expression value in doxycycline-treated cells versus untreated controls from 3 independent biological replicates; **p-student**, p-value of the corresponding fold-change value using a Student's *t*-test (within group).
(XLSX)

S6 Table **Primers used for this work and respective sequences.**
(DOCX)
