# Supplementary figures and images for: Targeted Changes of the Cell Wall Proteome Influence Candida albicans Ability to Form Single- and Multi-strain Biofilms
Source: PLoS Pathog. 2014 Dec 11;10(12):e1004542. doi: 10.1371/journal.ppat.1004542 (PMC4263760; doi:10.1371/journal.ppat.1004542)

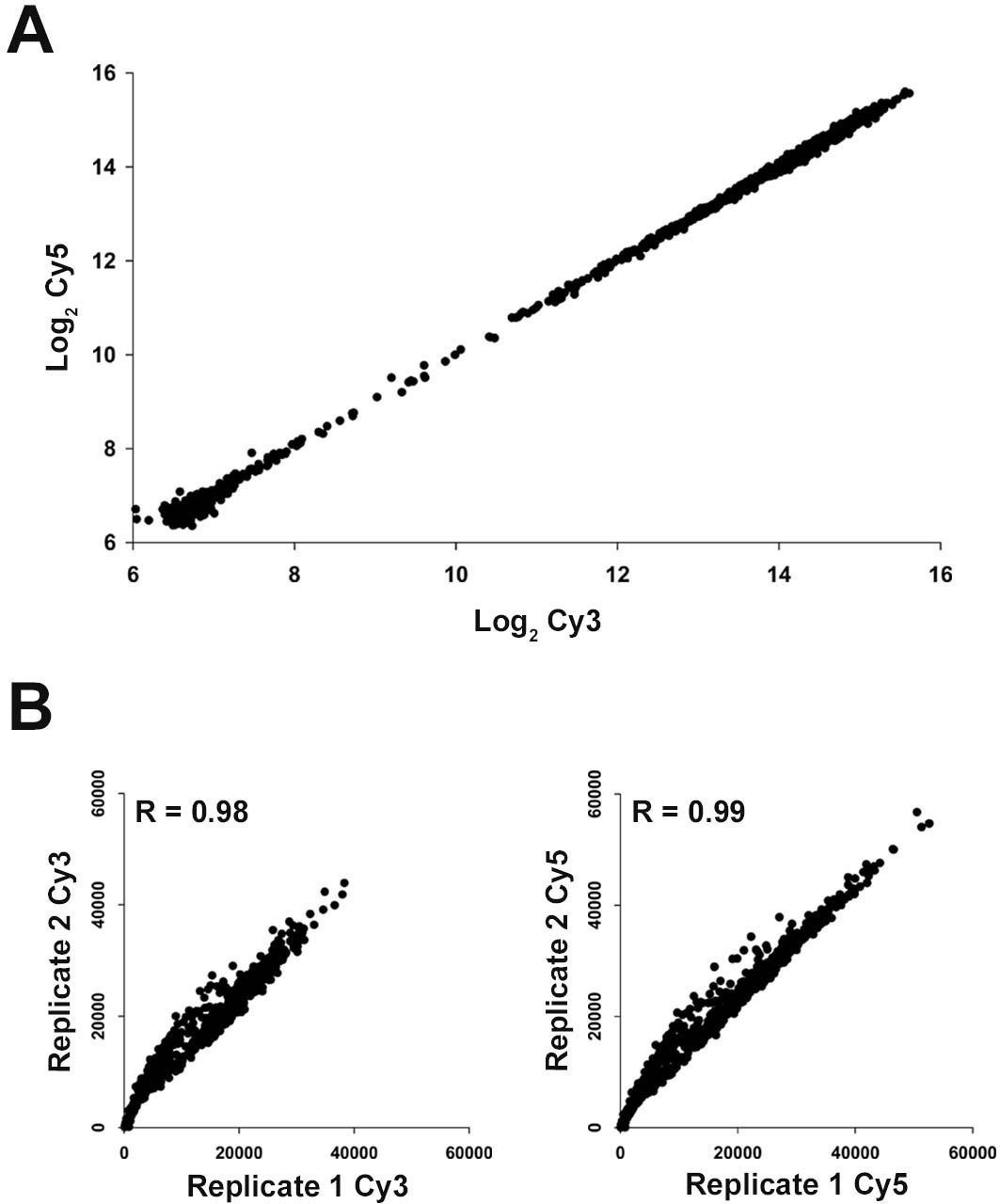

Supplement: S1 Figure — Validation of barcode detection by microarrays. Genomic DNA was extracted from two independent aliquots of the pooled strains (40 OD units each), followed by PCR-amplification of the barcodes, indirect differential fluorescent dye labeling of the pool-aliquot pair (Cy5 vs. Cy3) and hybridization to barcode microarrays (see materials and methods). (A) Signal scatter plot showing that signal intensities of either the negative control spots (background) or the unused tags were ranging within the log2-transformed values of 6–8. This interval also included ∼10% of strain tags, suggesting that the corresponding strains were underrepresented in the pool or that these tags had low hybridization efficiency. (B) Reproducibility of tag detection. The above-described experiment was performed twice independently and Pearson correlation coefficient (R) of the background-corrected raw signals was calculated for each channel. R coefficient values were >0.98, indicating that tag detection was highly reproducible. (JPG) [file ppat.1004542.s001.jpg]

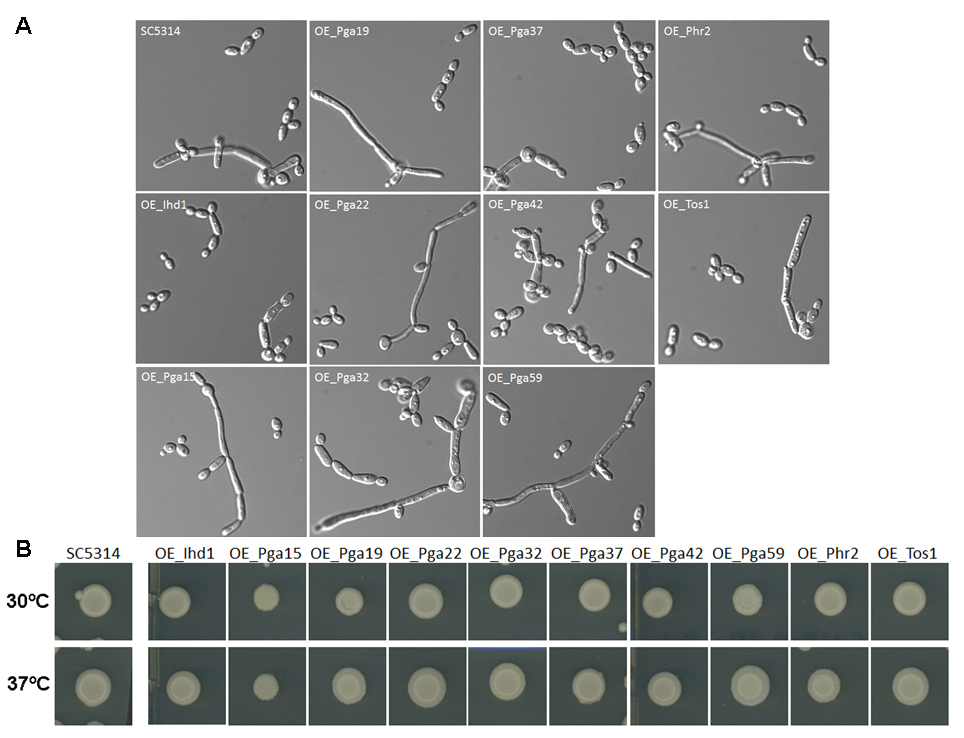

Supplement: S2 Figure — Phenotypic examination of the strains overexpressing the set of genes identified in the competitive overexpression screen during biofilm development. (A) Microscopic examination (40× magnification) of strains overexpressing IHD1/PGA36, PGA15, PGA19, PGA22, PGA32, PGA37, PGA42, PGA59, PHR2, and TOS1 together with the wild-type control SC5314 during growth in GHAUM liquid medium at 37°C in the presence of doxycycline. (B) Single colonies from the same strains were also grown on solid GHAUM medium at both 30°C and 37°C to test for growth rate alterations. (JPG) [file ppat.1004542.s002.jpg]

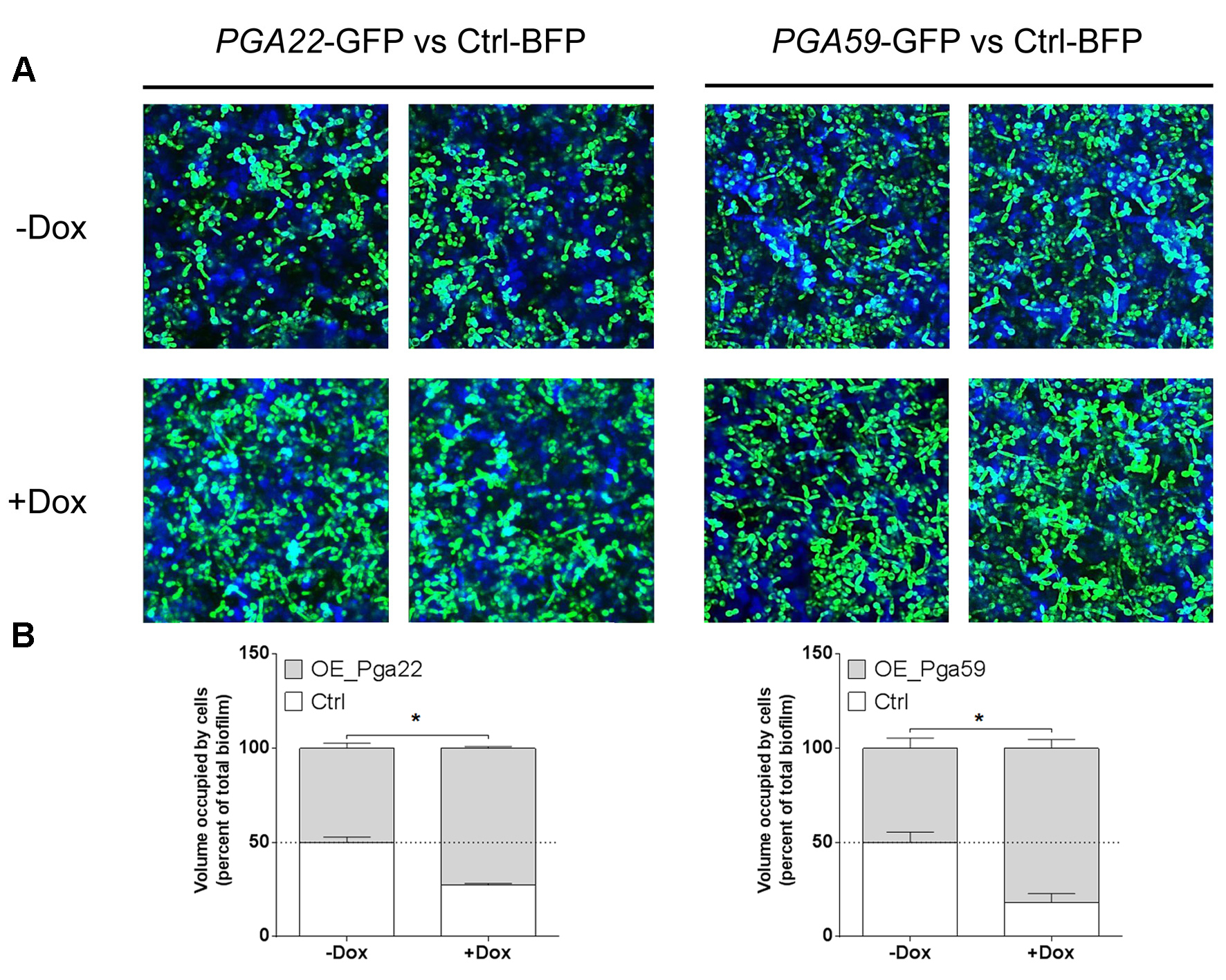

Supplement: S3 Figure — Confocal microscopy-acquired fluorescence images of mature biofilms made of GFP-labeled PGA22 or PGA59 overexpression strains in a 1∶1 mixture with BFP-labeled control parental strain. (A) Biofilms were developed for 40 h using as an inoculum a 1∶1 mixture of a BFP-expressing control strain and either a GFP-expressing PGA22-overexpressing strain (left panels) or a GFP-expressing PGA59-overexpressing strain (right panels) in the absence (-Dox, upper panels) or presence (+ Dox, bottom panels) of 50 µg.mL−1 doxycycline followed by acquisition of fluorescence intensity images using a confocal microscope. (B) Volume occupied by cells expressing GFP or BFP was quantified using Volocity software, and relative percentage of each strain is represented in the absence (-Dox) or presence (+ Dox) of 50 µg.mL−1 doxycycline. Data was averaged for 2 replicates, from two independent experiments, and standard error of means are shown; Student's t-tests were performed and results are represented on top of each graph (asterisk); * p≤0.05. (JPG) [file ppat.1004542.s003.jpg]

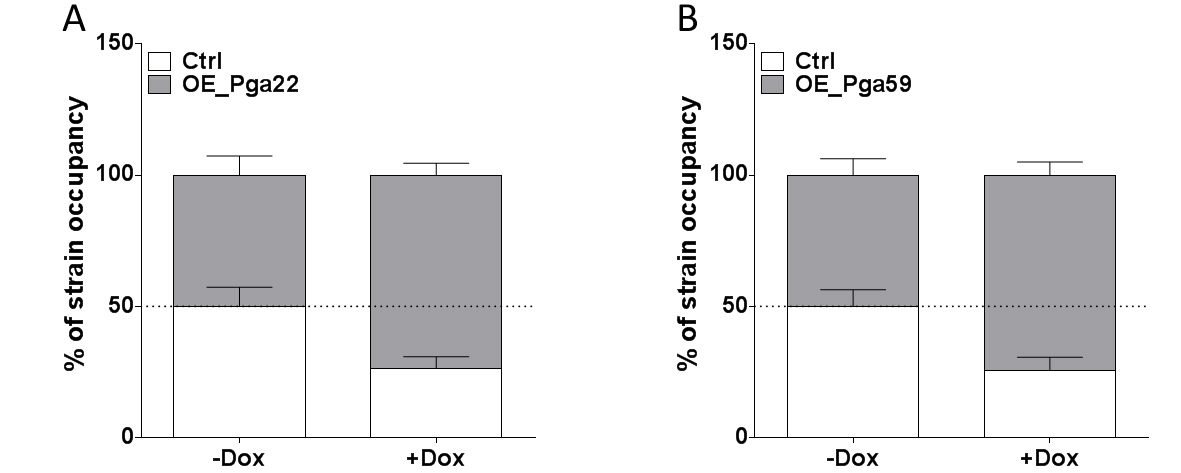

Supplement: S4 Figure — Higher occupancy of strains overexpressing PGA22 and PGA59 in a mixed biofilm formed with a wild-type strain. Biofilms were developed for 40 h using as an inoculum a 1∶1 mixture of a GFP-expressing control strain and either a BFP-expressing PGA22-overexpressing strain (A) or a BFP-expressing PGA59-overexpressing strain (B) in the absence (-Dox) or presence (+ Dox) of 50 µg.mL−1 doxycycline. The abundance of each strain in the mixed biofilm was quantified using qPCR on the GFP and BFP genes. Data averaged for 3 replicates and standard error of means are shown. (JPG) [file ppat.1004542.s004.jpg]

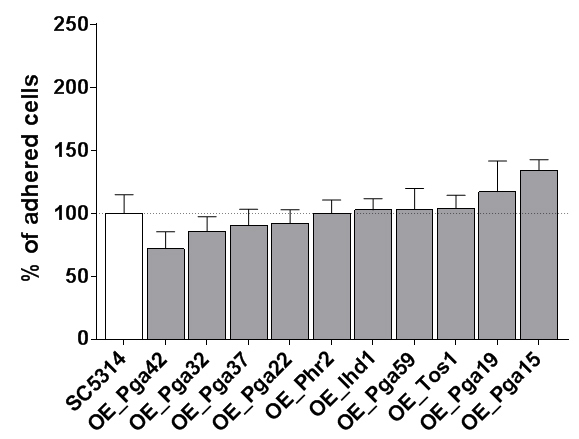

Supplement: S5 Figure — Uninduced overexpression strains for selected cell wall protein genes do not show alteration in adherence to Thermanox. Adherence of the overexpression strains to Thermanox was quantified following growth in the absence of doxycycline and normalized using the wild-type strain SC5314 as a control. Student's t-tests were performed on 10 pictures for each strain and did not reveal significant differences with the wild-type control. (JPG) [file ppat.1004542.s005.jpg]

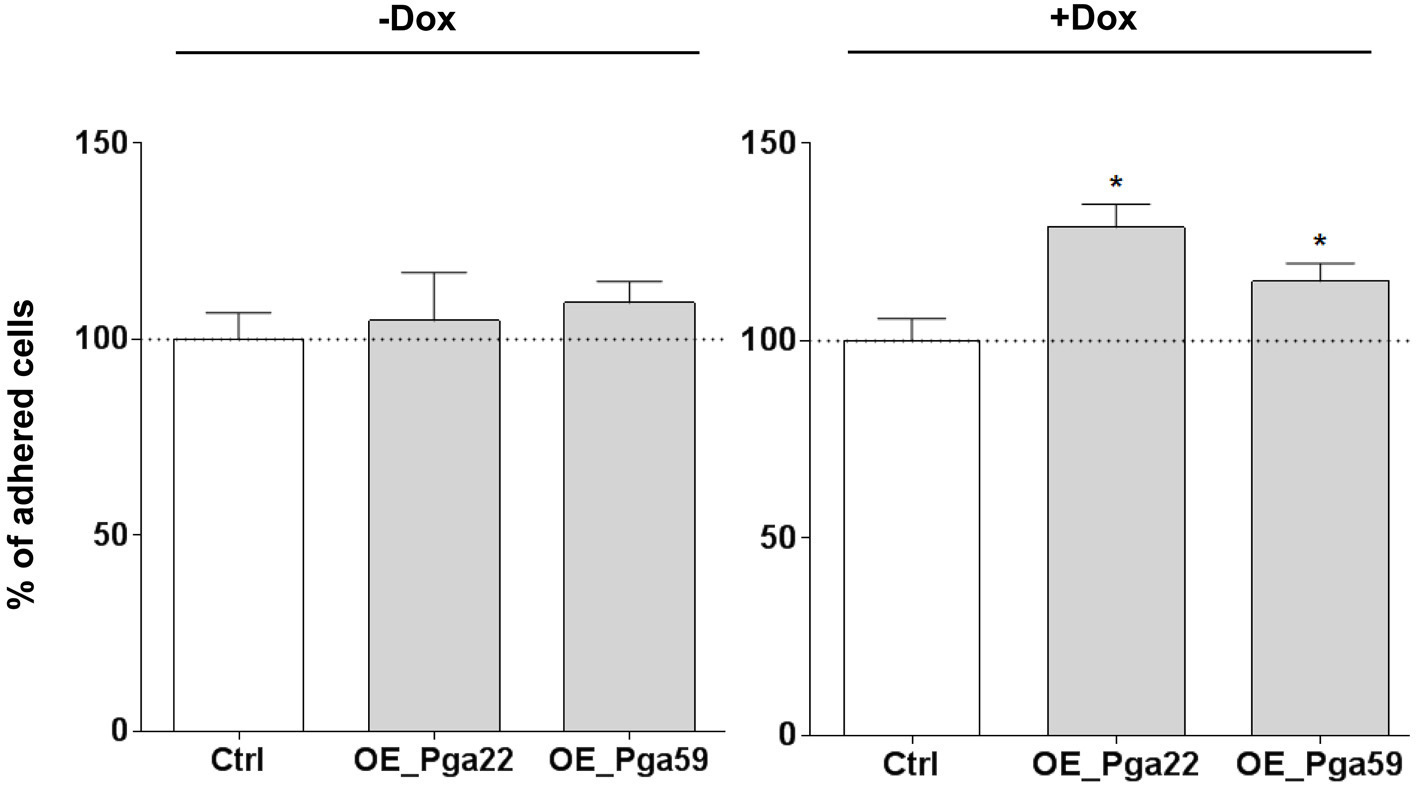

Supplement: S6 Figure — Overexpression of PGA22 and PGA59 increases cell adherence to polystyrene substrate. Adherence of the indicated overexpression strains (OE_Pga22, PGA22 overexpression; OE_Pga59, PGA59 overexpression) to polystyrene substrate was quantified following growth of strains in a microtiter plate, during 30 min, in the absence (-Dox) or presence (+ Dox) of 50 µg.mL−1 doxycycline. Data were normalized using as a reference the adherence shown by each strain in the absence of overexpression. Statistical tests (Student's t-tests) were performed on at least 10 pictures for each strain and results are represented on top of each bar (* p≤0.05). (JPG) [file ppat.1004542.s006.jpg]

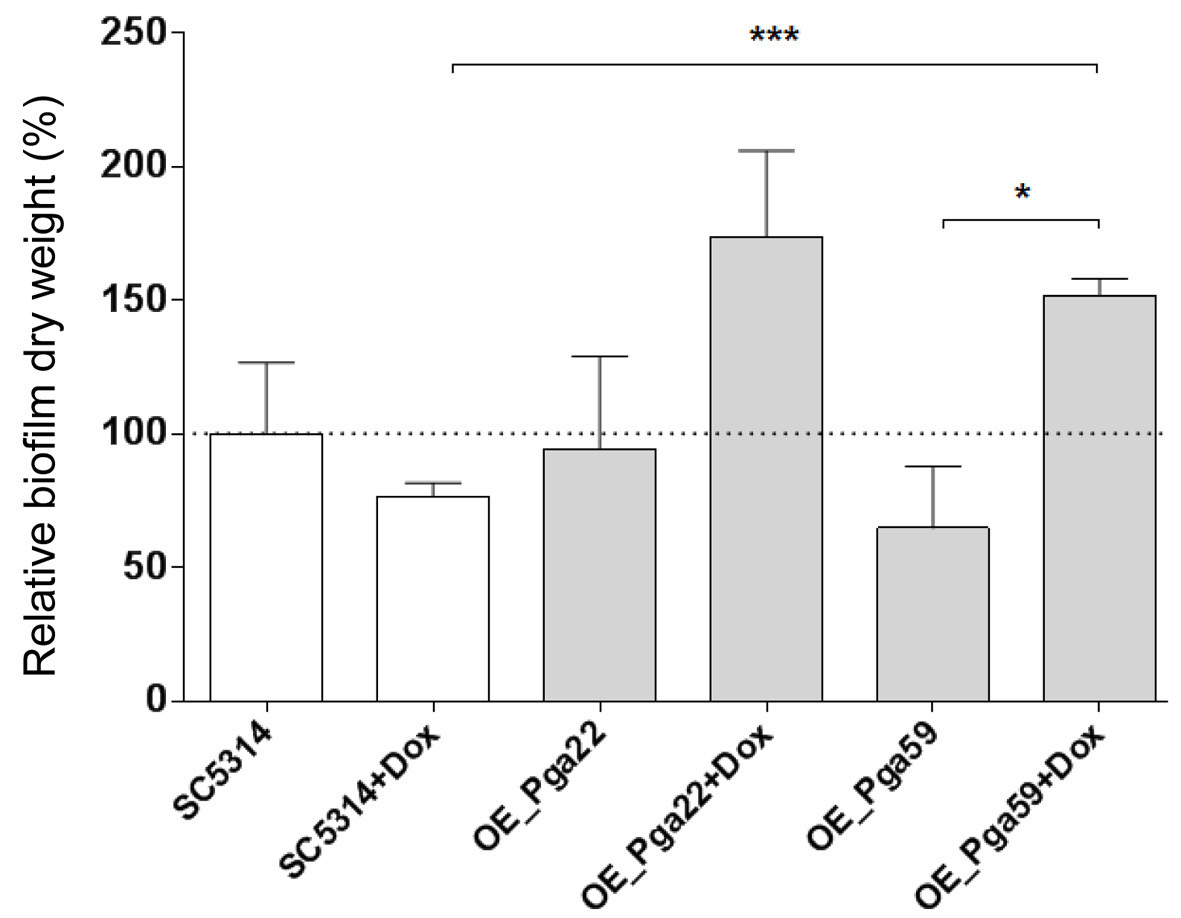

Supplement: S7 Figure — Effect of overexpressing PGA22 and PGA59 on biofilm formation under static growth conditions. Strains overexpressing PGA22 (OE_Pga22) or PGA59 (OE_Pga59) and strain SC5314 (wilde-type control) were individually grown in 6-well plates to form biofilms in the absence (-Dox) or presence (+ Dox) of doxycycline, with one washing step after adherence, followed by another wash after biofilm growth. Biofilms were recovered and dry weight obtained by filtration. Filters were left for 3 days at 65°C and weighed afterwards. Graphs are normalized for the WT strain's biofilm biomass. Data originate from two independent experiments, with at least n = 3 biological replicates (*, P≤0.05 from comparison between Pga59-Dox and Pga59+ Dox; ***, P≤0.001 from comparison between Pga59+ Dox and SC5314+ Dox using a two-tailed Student's t-test). (JPG) [file ppat.1004542.s007.jpg]

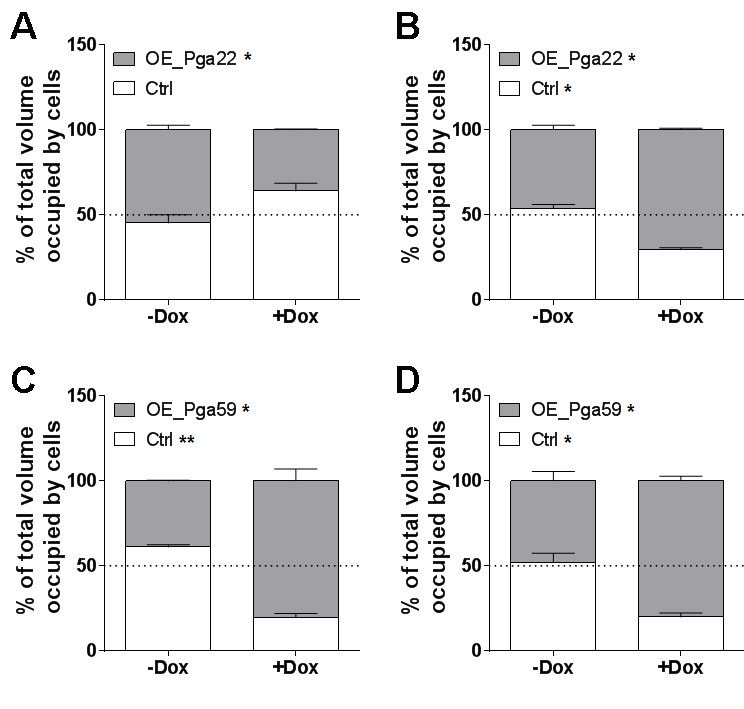

Supplement: S8 Figure — Quantification of strain abundance of GFP-labeled PGA22 - or PGA59 -overexpressing strains relative to BFP-labeled control strain in the lower versus upper layer of mature mixed-strain biofilms. Biofilms were developed for 40 h using as an inoculum a 1∶1 mixture of a GFP-labeled PGA22-overexpressing (A, B) or a GFP-labeled PGA59-overexpressing (C, D) strains relative to BFP-labeled control strain in the absence (-Dox) or presence (+ Dox) of 50 µg.mL−1 doxycycline followed by quantification of confocal microscopy-acquired GFP and BFP fluorescence signals within the bottom (A, C) and upper (B, D) layers of the mature biofilm (see Materials and Methods for details). *, P≤0.05; **, P≤0.01 using a two-tailed Student's t-test. Data was averaged for 2 replicates, from two independent experiments. (JPG) [file ppat.1004542.s008.jpg]

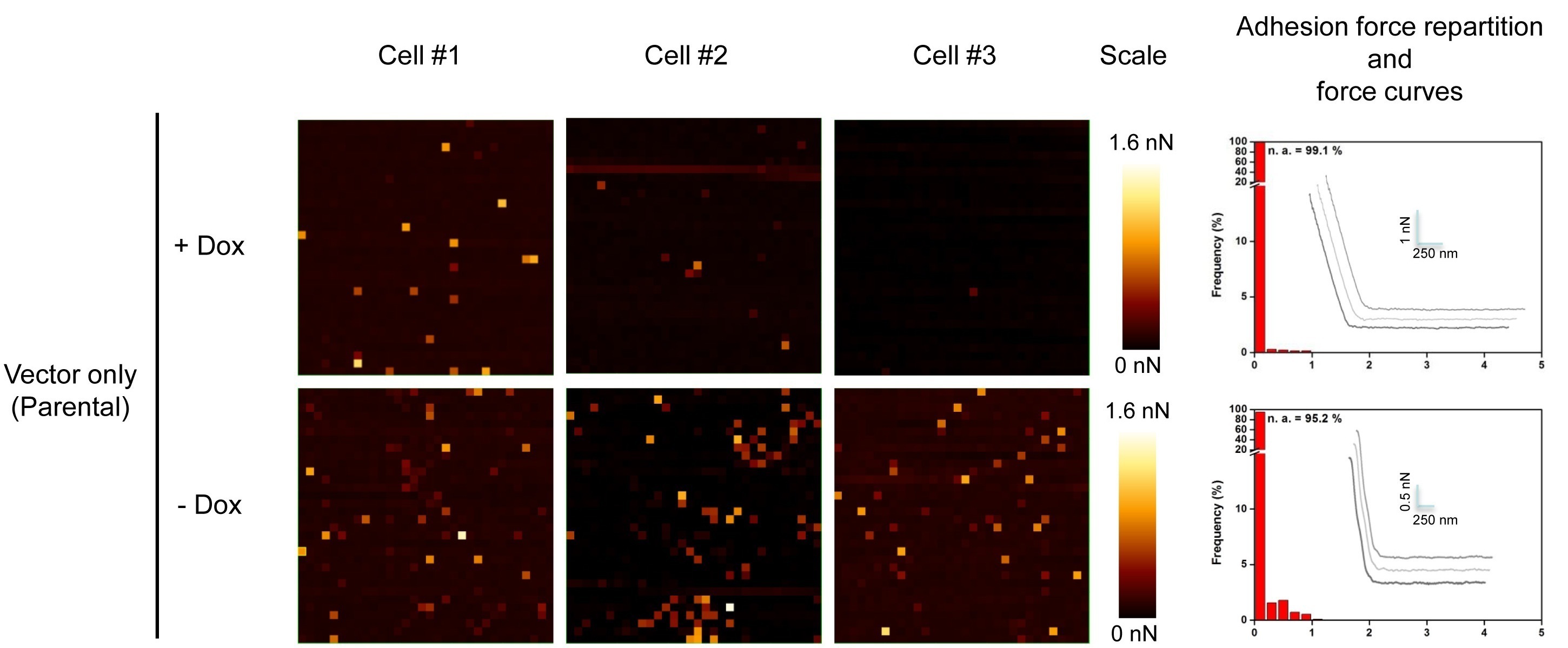

Supplement: S9 Figure — AFM-derived adhesion measurements in the control strain CEC3785. Adhesion maps in three independent C. albicans cells (Cells #1–3, each analyzed area covers 1×1 µm2) recorded on the control strain (CEC3785) with empty vector, treated (+ Dox) or not (-Dox) with 50 µg.mL−1 doxycycline during 16 h in YPD. Adhesion scales are shown (Scales; bright yellow, maximum at 1.6 nN; dark red, minimum at 0.0 nN). The corresponding histograms representing the adhesion force repartition (red bars) and representative force curves (grey lines; scales are indicated with light blue bars) are shown at the right of each panel. (JPG) [file ppat.1004542.s009.jpg]

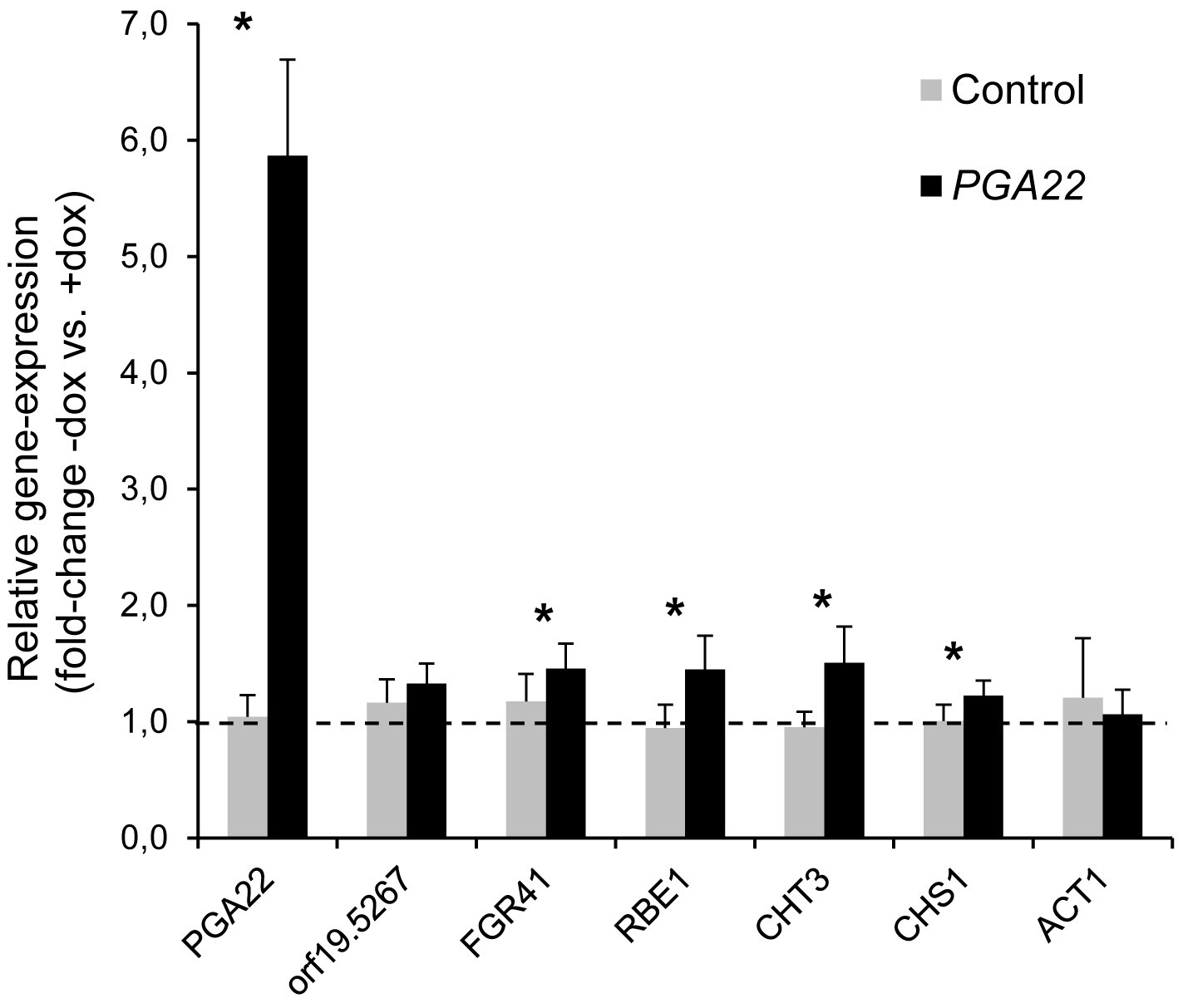

Supplement: S10 Figure — RT-qPCR analysis of selected genes whose expression was induced upon PGA22 overexpression. Quantitative real-time RT-PCR analysis of PGA22, ORF19.5267, FGR41, RBE1, CHT3, CHS1 and ACT1 (as a control). Bars indicate the relative changes in RNA expression of the indicated genes in doxycycline-treated samples versus untreated for the BWP17 parental strain carrying the empty vector (Control, light gray bars) and the derived PGA22 overexpression strain (black bars). Asterisks denote statistical significance by two-tailed Student's t-test (P≤0.05) between the doxycycline-treated and untreated samples. Error bars denote standard deviations. The assay was performed using 3 independent experiments performed on different days, each using two biological replicates (assumed as n = 6 in total). (JPG) [file ppat.1004542.s010.jpg]
