# Supplementary material for: Targeted Changes of the Cell Wall Proteome Influence Candida albicans Ability to Form Single- and Multi-strain Biofilms
Source: PLoS Pathog. 2014 Dec 11;10(12):e1004542. doi: 10.1371/journal.ppat.1004542 (PMC4263760; doi:10.1371/journal.ppat.1004542)
Supplement: S4 Table — Parental and deletion strains used in the course of this work and respective genotypes. References for the indicated strains are listed at the bottom of S4 Table. (DOCX) [file ppat.1004542.s014.docx]

Table S4. Parental and deletion strains used in the course of this work and respective genotypes.

| **Strain** | **Genotype** | **Reference** |
| --- | --- | --- |
| *Candida albicans*  BWP17 | *ura3∆::λimm434/ura3∆::λimm434 arg4∆::hisG*/*arg4∆::hisG* *his1∆::hisG*/*his1∆::hisG* | [[1](#_ENREF_1)] |
| CEC3785 | *ura3∆::λimm434/ura3∆::λimm434 arg4∆::hisG*/*ARG4* *his1∆::hisG*/*HIS1 ADH1/adh1::P_TDH3_-carTA::SAT1 RPS1/RPS1::* CIp10-P*_TET_*-GTW | This study |
| BWP17-GFP | *ura3∆::λimm434/ura3∆::λimm434 his1∆::hisG/HIS1 arg4∆::hisG/ARG4 RPS1/RPS1::*CIp10*-*P*_TDH3_*-GFP | This study |
| BWP17-mCherry | *ura3∆::λimm434/ura3∆::λimm434 his1∆::hisG/HIS1 arg4∆::hisG/ARG4 RPS1/RPS1::*CIp10*-*P*_ADH1_*-mCherry | This study |
| CAI4 | *ura3∆::λimm434/ura3∆::λimm434* | [[2](#_ENREF_2)] |
| SN76 | *arg4∆/arg4∆ his1∆/his1∆ ura3∆::λimm434/ura3∆::λimm434 iro1∆::λimm434/iro1∆::λimm434* | [[3](#_ENREF_3)] |
| SN148 | *arg4∆/arg4∆ leu2∆/leu2∆ his1∆/his1∆ ura3::λimm434/ura3∆::λimm434 iro1∆::λimm434/iro1∆::λimm434* | [[3](#_ENREF_3)] |
| SN152 | *his1*Δ*/his1*Δ *leu2*Δ */leu2*Δ *arg4*Δ */arg4*Δ *URA3/ura3*Δ*::*λ*imm434, IRO1/iro1*Δ*::*λ*imm434* | [[3](#_ENREF_3)] |
| CEC1121 | SN148 *GAL1/gal1::LEU2 ADH1/adh1::P_ADH1_-cartTA::SAT1::P_TET_-caGFP* | This study |
| CEC1429 | CAI4 *ADH1/adh1::P_ADH1_-cartTA::SAT1::P_TET_-caGFP* | This study |
| CEC2908 | *ura3∆::λimm434/ura3∆::λimm434 his1∆::hisG/HIS1 arg4∆::hisG/ARG4 ADH1/adh1::P_TDH3_-carTA::SAT1* | [[4](#_ENREF_4)] |
| CEC3781 | SN76 *his1∆/HIS1 ADH1/adh1::P_TDH3_-carTA::SAT1 Ca21chr4_C_albicans_SC5314:473390 to 476401∆::P_TDH3_-GFP-CaARG4* | This study |
| CEC3783 | SN76 *arg4∆/ARG4 ADH1/adh1::P_TDH3_-carTA::SAT1 Ca21chr4_C_albicans_SC5314:473390 to 476401∆::P_TDH3_-BFP-HIS1* | This study |
| *∆∆pga22* | BWP17 *pga22∆::ARG4/pga22∆::HIS1 RPS1/RPS1::*CIp10 | This study |
| *∆∆pga22*-GFP | BWP17 *pga22∆::ARG4/pga22∆::HIS1 RPS1/RPS1::*CIp10*-*P*_TDH3_*-GFP | This study |
| *∆∆pga22*-mCherry | BWP17 *pga22∆::ARG4/pga22∆::HIS1 RPS1/RPS1::*CIp10*-*P*_ADH1_*-mCherry | This study |
| *∆∆pga59* | BWP17 *ura3∆::λimm434* URA3 pga59Δ: : HIS1/pga59Δ: : ARG4 | [[5](#_ENREF_7)] |
| *Saccharomyces cerevisiae* | |  |
| BY4742  VIF205 | *MATα his3Δ1 leu2Δ0 lys2Δ0 ura3Δ0*  BY4742/pBC542 | [[6](#_ENREF_9)]  [7] |

1. Wilson RB, Davis D, Mitchell AP (1999) Rapid hypothesis testing with *Candida albicans* through gene disruption with short homology regions. J Bacteriol 181: 1868-1874.

2. Fonzi WA, Irwin MY (1993) Isogenic strain construction and gene mapping in *Candida albicans*. Genetics 134: 717-728.

3. Noble SM, Johnson AD (2005) Strains and strategies for large-scale gene deletion studies of the diploid human fungal pathogen *Candida albicans*. Eukaryot Cell 4: 298-309.

4. Chauvel M, Nesseir A, Cabral V, Znaidi S, Goyard S, et al. (2012) A versatile overexpression strategy in the pathogenic yeast *Candida albicans*: identification of regulators of morphogenesis and fitness. PloS One 7: e45912.

5. Moreno-Ruiz E, Ortu G, de Groot PW, Cottier F, Loussert C, et al. (2009) The GPI-modified proteins Pga59 and Pga62 of *Candida albicans* are required for cell wall integrity. Microbiology 155: 2004-2020.

6. Brachmann CB, Davies A, Cost GJ, Caputo E, Li J, et al. (1998) Designer deletion strains derived from *Saccharomyces cerevisiae* S288C: a useful set of strains and plasmids for PCR-mediated gene disruption and other applications. Yeast 14: 115-132.

7. Monniot C, Boisramé A, Da Costa G, Chauvel M, Sautour M, et al. (2013) Rbt1 Protein Domains Analysis in *Candida albicans* Brings Insights into Hyphal Surface Modifications and Rbt1 Potential Role during Adhesion and Biofilm Formation. PloS one 8: e82395.
