# Supplementary material for: Targeted Changes of the Cell Wall Proteome Influence Candida albicans Ability to Form Single- and Multi-strain Biofilms
Source: PLoS Pathog. 2014 Dec 11;10(12):e1004542. doi: 10.1371/journal.ppat.1004542 (PMC4263760; doi:10.1371/journal.ppat.1004542)
Supplement: S6 Table — Primers used for this work and respective sequences. (DOCX) [file ppat.1004542.s016.docx]

Table S5. Primers used for this work and respective sequences.

| **Name** | **Sequence (5' - 3')** |
| --- | --- |
| CipSAC2-UP-2 | CCTATTAGTCGGGATCTAGG |
| CipSAC2-DWN-2 | CACTATAGGGCGAATTGG |
| BFPpFwd | ATGGGAGGCATTCACTGAG |
| BFPpRev | GCTATCAAGTGTGAACCTCCT |
| GFPpFwd | TCCACTCAATCTGCCTTATCC |
| GFPpRev | CATGGGTAATACCAGCAGCA |
| TEF3-F | CAAGAAATGTCCATCTGCTCAATC |
| TEF3-R | TGTTTAGTTTTAACCCCTTCCAAGA |
| mCherry2-FWD | GTAGACCATATGAAGGTACTCAAAC |
| mCherry2-REV | TAACAGTAACAACACCACCATC |
| mCherry-FWD | GTTACTAAAGGTGGTCCATTACCAT |
| mCherry-REV | GAGTAACAGTAACAACACCACCATC |
| GFP.RT.fw | ggctgacaaacaaaagaatgg |
| GFP.RT.rv | gaccatcaccaattggagta |
| ACT1-FWD | TATGAAAGTTAAGATTATTGCTCCACCAGAAA |
| ACT1-REV | GGAAAGTAGACAATGAAGCCAAGATAGAAC |
| CHT3-FWD | AGGTGGTGCTGCTGGATCTTATGG |
| CHT3-REV | TGAGCAAATTGTTTGGCAGTGGCA |
| orf19.5267-FWD | CCACTACACCTTATGTCACTGCTA |
| orf19.5267-REV | TTATCATCACTACTTGAGGGATCAG |
| FGR41-FWD | CCAGGTATACTGCTACTGTCACTTC |
| FGR41-REV | CTTAGCAACAGGAGTTGTGGTAGT |
| RBE1-FWD | CTACTAGCTCCGTCAACTCATTAAC |
| RBE1-REV | GAGTGCTCTCTTGACATTATGTTCT |
| CHS1-FWD | GTTGGTGGCAAAGCAGGTAAC |
| CHS1-REV | CTCGGTTCTGGTCAACACTTTTC |
| PGA22-FWD | TCCAACCACTACCCATGAGAATACA |
| PGA22-REV | TTGTTGTTGTAAGTGCTGGTCCTTC |
| PGA22-GTW-fwd | GGGGACAAGTTTGTACAAAAAAGCAGGCTTGATGAAGTATTCGACTTTAGCTTGGCTTGTT |
| PGA22∆Cter-rev | GGGGACCACTTTGTACAAGAAAGCTGGGTGTTTCGGTTCCGGTGCCTGTTAAG |
| 3738J5DR | ATGTCATATCAAAGCATTGTTATTGTCCTATAGCAAGCTCTTGTCCTTTCCTTGTCTAGAAAAATCATGTATTCAAGGGATGTGAAGGCGGCTGTAAAGCTTTCCCAGTCACGACGTT |
| 3738J3DR | CTATAAGAACAATAAGGCAACCAAATATCCCAAAGATGCACCAGCAGCCAATGAGAAGGCTTCAGCAACAGTTGTTGTTGTAAGTGCTGGTCCTTCGGTTGTGGAATTGTGAGCGGATA |
